# Next-Generation Wastewater Treatment with Emerging 2D MOF-Derived Photocatalysts

**DOI:** 10.1021/acsomega.5c08223

**Published:** 2025-11-21

**Authors:** Naveen Thakur, Anchal Panwar, Vatika Soni, Pankaj Raizada, Pardeep Singh, Aftab Aslam Parwaz Khan, Van-Huy Nguyen, Chinh Chien Nguyen, Seok Joo Yang, Quyet Van Le, Hyojung Kim, Sonu Sonu

**Affiliations:** † School of Advanced Chemical Sciences, 375605 Shoolini University, Solan 173229, India; ‡ Center of Excellence for Advanced Materials Research, 37848 King Abdulaziz University, P.O. Box 80203, Jeddah 21589, Saudi Arabia; § Centre for Herbal Pharmacology and Environmental Sustainability, Chettinad Hospital and Research Institute, 77239 Chettinad Academy of Research and Education, Kelambakkam 603103, Tamil Nadu, India; ∥ Center for Advanced Chemistry, Institute of Research and Development, 35006 Duy Tan University, Danang 550000, Vietnam; ⊥ Faculty of Natural Sciences, Duy Tan University, Danang 550000, Vietnam; # Department of Chemical Engineering, 26720 Gyeongsang National University, Jinju 52828, Republic of Korea; ¶ College of Space and Aeronautics, Gyeongsang National University, Jinju 52828, Republic of Korea; ∇ Department of Semiconductor Systems Engineering, Sejong University, 209, Neungdong-ro, Gwangjin-gu, Seoul 05006, Republic of Korea

## Abstract

Metal–organic frameworks (MOFs) have emerged as a significant class of heterogeneous photocatalytic materials owing to their structural tunability and high porosity. Among them, two-dimensional MOFs (2D MOFs) have gained increasing attention for various photocatalytic applications due to their ultrathin layered structures, large surface areas, abundant exposed active sites, and enhanced charge migration characteristics. Compared to their 3D counterparts, 2D MOF-derived nanocomposites often exhibit improved photocatalytic efficiency. Recent studies have emphasized the rational design of 2D MOFs through precise control of metal nodes, organic linkers, and structural morphology to tailor their photocatalytic properties for specific targets. This review provides a concise overview of fabrication and postsynthetic modification strategies to enhance the photocatalytic performance of 2D MOF-derived nanocomposites. Practical applications, including environmental remediation, are discussed to demonstrate their real-world potential. Furthermore, the review highlights the intrinsic relationship between 2D MOF structure and photocatalytic behavior, along with current challenges such as structural instability, scalability, and limited visible-light absorption. Finally, key perspectives and future directions are proposed to guide the development of robust, efficient, and sustainable 2D MOF-based photocatalytic materials.

## Graphical abstract

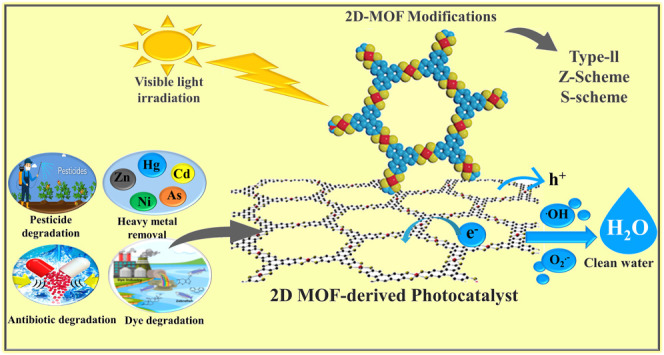

## 1 Introduction

In the modern era, the expansion of industries has bolstered the global economy, driving innovation and economic progress.^1^ The recent industrial surge has raised significant concerns regarding environmental and human health, particularly due to widespread contamination of water sources and the degradation of soil quality. The presence of toxic pollutants in wastewater poses a serious environmental threat. High concentrations of these hazardous industrial pollutants, such as heavy metals, textile dyes, pharmaceutical residues, and antibiotics, represent a major challenge to both environmental sustainability and public well-being.^2,3^ Conventional wastewater treatment methods are effective at removing certain pollutants; however, they struggle with complex and resilient contaminants. Therefore, there is a need to develop innovative solutions that can efficiently degrade these pollutants while promoting environmental sustainability, cost-effectiveness, and energy efficiency. This will lead to the purification of contaminants and help neutralize their adverse effects on the environment.^1,4^ Over the past few decades, various techniques, including adsorption,^5–7^ electrocatalysis,^8^ photoelectrocatalysis,^9^ biodegradation,^10^ electrochemical treatment,^11^ photocatalysis,^12^ and advanced oxidation processes,^13^ have been employed to eliminate and degrade pollutants across various media, thereby setting the environment free. Out of these, the one with an eco-friendly nature and remarkable ability to transform pollutants into nontoxic substances is the photocatalysis.^3^ Among these, photocatalysis stands out not only for its sustainability but also for its fundamental mechanism, which involves light-induced electron transitions that drive redox reactions over the catalyst.

In the photocatalytic process, electrons (e^–^) are migrated toward conduction band (CB) from the valence band (VB) under the influence of light radiations having energy greater than the band gap between VB and CB of the material, thus resulting in the generation of holes (h^+^) in the VB while free e^–^ in the CB.^14,15^ These e^–^–h^+^ pairs relocate to the surface of the material and interact with the dissolved oxygen and absorbed water molecules, leading to the generation of ROS (reactive oxygen species), including superoxide (O_2_^•–^) and hydroxyl (^•^OH).^15–17^ Metal organic frameworks (MOFs) act as photocatalysts, utilizing light energy to trigger chemical reactions and thus emerged as an innovative class of materials, demonstrating exceptional potential in wastewater treatment.^4,18^ MOFs are highly porous crystalline structures with a larger surface area and adjustable chemical properties formed by the coordination of inorganic nodes (metal clusters) with organic linkers (ligands).

Preparing metal–organic frameworks (MOFs) with numerous combinations results in nanocomposites with unique properties, such as quantum materials, metal oxides, enzymes, polymers, or carbon-based materials. Each combination can target specific functions, enhancing the limits and capabilities of MOF materials.^19^ (a) Porous Coordination Networks (PCNs): PCNs are fascinating with their 3D cage-like architecture with holes, including PCN-222, PCN-333, PCN-57, and PCN-224. The versatility in applications, like PCN-222, a DNA detecting sensor synthesized by Ling et al., plays a crucial role in genomics and diagnostics.^20^ In another report, PCN-134­(Zn)-2D nanosheets were synthesized by and utilized as a photocatalytic composite for an open-air PET-RAFT polymerization.^21^ Also, Li et al. employed PCN-134 (2D MOF) for the oxidation of ranitidine into less toxic products.^22^ (b) Isoreticular MOFs: These MOFs are fabricated by coordination of [Zn_4_O]^6+^ as a secondary building unit with a succession of aromatic carboxylates, giving rise to octahedral crystalline microporous materials. The creation of nanosheets from IRMOF-3 for the spotting of 2,4,6-trinitrophenol in wastewater by Zhu and colleagues showcases the precision and efficiency of these materials.^23,24^ (c) Zeolitic Imidazolate Frameworks (ZIFs): ZIFs resemble zeolites in structure and are synthesized by a combination of transition metals with imidazole derivatives. Generally, ZIF-based MOF composites are blessed with higher stability, higher pore size, and resistance toward heat.^25,26^ For example, a novel 2D ZIF-8 was fabricated via bulk layered ZIF-8 using a series of treatments such as freezing processes using liquid nitrogen and sonication in deionized water.^27^ Thus, the prepared 2D ZIF-8 demonstrated effective dye degradation. Other examples are ZIF-7, ZIF-67, ZIF-8, ZIF-90, ZIF-71, etc. Out of which, Pan and colleagues synthesized ZIF-8, which is used for the detection of HIV-1 DNA, and the properties like larger surface area, least cytotoxic, sensitivity toward acids, and larger pore size, make it a step ahead than other MOFs of the same category.^28,29^ (d) Porous Coordination Polymers (PCPs): PCPs are highly porous materials that offer several uses, including medication delivery, gas storage, catalysis, and separation. These are fascinating structures created by the self-assembly of organic linkers like carboxylic acid or pyridine with transition metals. For example, PCP-5, a 2D layered framework, demonstrates superior photocatalytic activity in degrading dyes along with the reusable property of up to five cycles.^30^ Also, Özcan and team fabricated a novel 2D coordination polymer PCP-10 using the solvothermal method via tetra carboxylate and N-donor terpyridine ligands.^31^ The as-prepared nanocomposite demonstrated 2D sheet-like morphology and was utilized for the degradation of organic dyes. (e) MOF with Infinite Coordination Polymers (MILs): A dicarboxylic acid ligand is combined with various transition metal ions, including Fe, Cr, Ti, and Al, or metal oxides, to create MILs. Consequently, MILs are produced by integration of transition metal ions with an organic linker that possesses two –COOH groups. For instance, MIL-88­(Fe) was successfully synthesized by Vigneshwaran and colleagues, showing a 2D thin-layered sheet-like morphology.^32^ Another example includes 2D NH_2_-MIL-125­(Ti) nanosheets (MTNs) prepared using the solvothermal method.^33^ (f) UiO-MOFs: In photodegradation work, UiO-66, UiO-67, UiO-68, and UiO-69 are the most often used UiO-MOFs, with UiO-66 being the most often active in its novel form or with different refinement.^34^ Lillerud and associates synthesized an UiO-MOF formulated as UiO-66­(Zr) for the first time by employing Zr6­(μ3-O)­4­(μ3-OH) and dicarboxylic acid as building units.^35^ In another study, Li et al. prepared two-dimensional Zr-MOFs formulated as UiO-67 NS using microwave radiation and benzoic acid and hydrochloric acid as modulating acids.^36^

Thus, MOF composites have increased structural variation because of their various dimensionalities, which include 0D, 1D, 2D, and 3D.^37^ Among them, 2D MOFs with exfoliated single layers provide several benefits, including readily accessible active sites for the substrates.^38,39^ Although wider band gap and limited redox activity of 2D MOFs along with low stability due to the hydrophilic nature of the metal center limit their practical applications.^40^ To overcome these drawbacks, various modification strategies have been employed, such as type-II-, Z-scheme-, and S-scheme-based heterojunctions.^41^ Researchers have been continuously improving the properties of 2D MOFs to boost their conductivity and electrocatalytic activity. (Scheme 1) illustrates a timeline showcasing significant research milestones and advancements in the use of pure 2D MOF photocatalysts for wastewater purification.

**Scheme 1:**
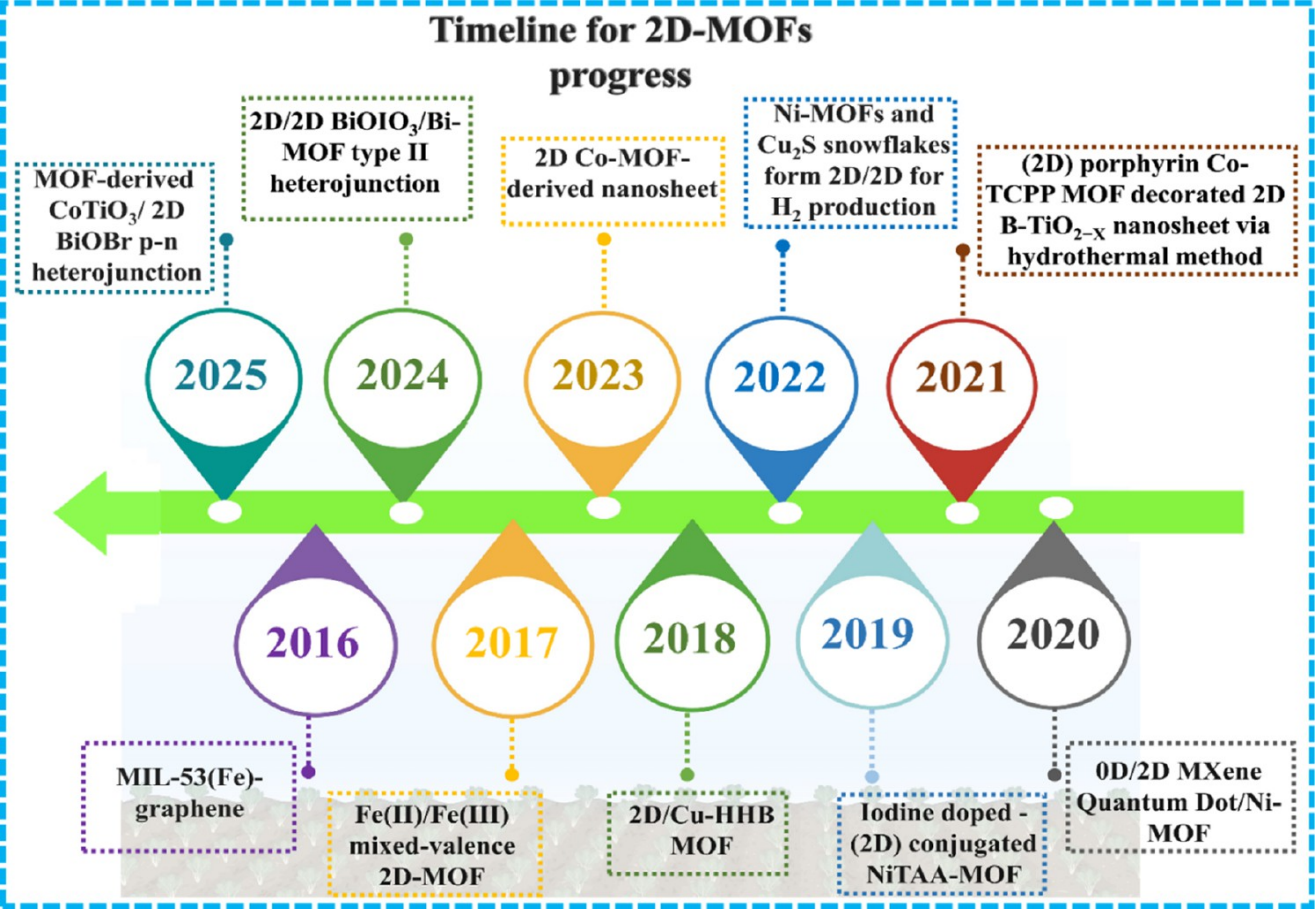
Timeline Showing the Evolution of 2D MOF-Based Photocatalysts for Wastewater Treatment

This review seeks to provide a comprehensive analysis of 2D MOF-derived photocatalysts and investigate how versatile they are in supporting the catalytic process of wastewater pollutant degradation. There are several existing works that discuss the practical applicability of these photocatalysts. However, no review that addresses the various fabrication processes of 2D MOF-based photocatalysts has been published.^42–44^ Additionally, our work explores the advanced structural and electronic modification strategies aimed at maximizing 2D MOF’s photocatalytic activity through heterostructure creation. Thus, the study evaluates the practical efficacy of 2D MOF-based catalysts and their applications through real-world case studies. This review intends to offer valuable facts regarding the potential of 2D MOF-derived photocatalysts in sustainable water purification and propose future research directions.

### 1.1 2D MOF

Extensive research has been conducted on 2D MOF nanosheets because of their remarkable physical and chemical properties, including their large specific surface area, high aspect ratio, extremely thin layers, chemical composition that can be altered, abundance of exposed unsaturated metal sites, and surface atomic structures that can be distinguished (Figure 1).^39^ As summarized in Table 1, 2D MOFs generally exhibit higher specific surface areas and enhanced photocatalytic performance compared to their bulk MOF counterparts, highlighting their potential in advanced catalytic applications. These benefits include increased stability in the presence of oxidative and reductive gases, thickness at the nanoscale, and resistance to severe reaction conditions such as elevated temperatures. The creation of a film due to 2D morphology facilitates light radiation penetration, increasing the nanocomposite’s capacity to capture light. Because of all these features, 2D MOFs are more effective and competent than their counterparts in the photocatalysis process. The remarkable properties with versatility and tunability make 2D MOFs exceptionally effective in facilitating catalytic reactions, particularly those associated with the breakdown of environmental pollutants. 2D MOFs with designable and modifiable frameworks enable precise decay of a variety of toxic contaminants by integrating specific active sites or groups, making bare 2D MOFs capable of selectively interacting and breaking down particular pollutants.^3,45–47^ 2D morphology is particularly well-suited for the formation of films and coatings on surfaces.^38^ Both surface adhesion and the percentage of contact area between the 2D MOFs and the substrate are increased by the 2D shape. For photocatalysis to be very effective in harvesting light, all of the photoresponsive units must be exposed to photons. When light penetration in opaque powders is restricted to a few microns, the formation of these films is extremely advantageous for photocatalysis. As a result, films that are as thick as the depth of light penetration must be prepared. Thus, given the growing significance of photocatalysis in the context of using sunlight as a major energy source, 2D MOFs seem more practical than other counterparts. In catalytic domains, the Fabrication of 2D MOF-derived nanocomposites and the study of the relationship between their performance and structure have been attributed to the outstanding achievements.^39^ The catalytic activity of 2D MOF-based materials may be precisely tuned by controlling the unsaturated metal nodes, functionalizing organic ligands by postsynthetic modification, and choosing a variety of ligands with conjugated structures or functional groups. Also, 2D MOFs can serve as scaffolds for encapsulating nanoparticles and incorporating single-atom catalysts. For example, 2D heterojunction Zn-MOF-NH_2_/Cu was prepared by Lu and team using facile reaction treatment.^48^ The synthesized nanocomposite exhibited excellent photocatalytic CO_2_ reduction capability attributed to highly exposed active sites and nearer e^–^ localization, which directly correlates with the 2D morphology of the nanomaterial. The series obtained on the basis of the activity of the photocatalyst was given as: 2D Zn-MOF-NH_2_/Cu > 2D Zn-MOF-NH_2_ > bulk Zn-MOF-NH_2_. This trend showed an enhanced impact of the 2D heterostructure on the photocatalytic performance than the bulk nanomaterial. Furthermore, the impression of 2D heterostructure on the photocatalyst activity can be seen in a study where Pt/Zr-TCPP­(Pd) nanosheets (NSs) exhibited superior H_2_ production.^49^ The enhanced performance is attributed to the faster charge uncoupling feature of Zr-TCPP­(Pd) NSs and high-speed e^–^ migration between PtNPs and Zr-TCPP­(Pd) NSs due to their fine-established interfacial contact. The result from the study also depicted the superiority of the prepared 2D nanocomposite over Pt/bulk Zr-TCPP­(Pd) with 5 times more performance.

**Figure 1:**
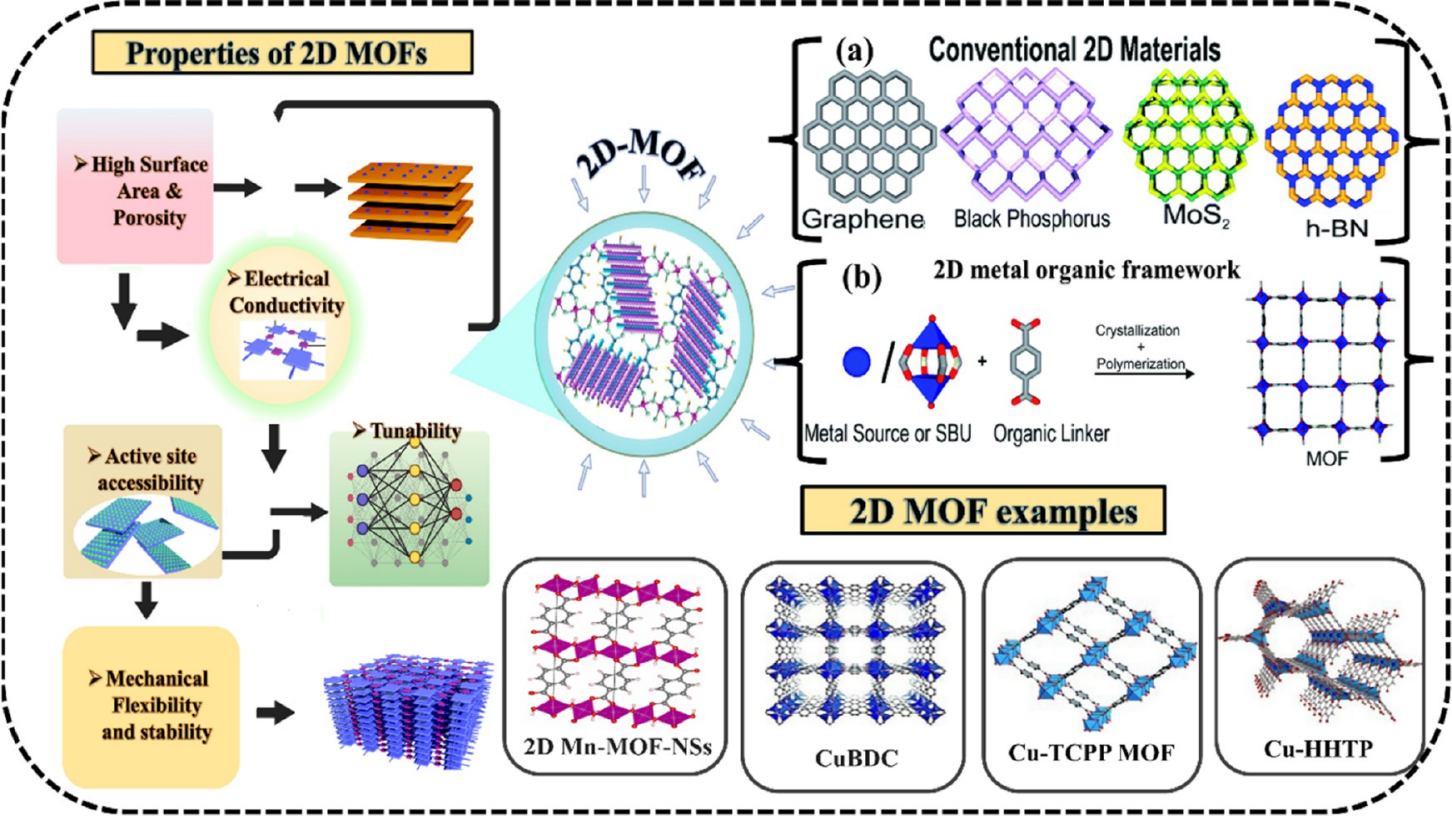
Basic properties and structure of different 2D MOFs, (a) conventional 2D materials and (b) 2D MOF; Adapted with permission from RSC, and Elsevier,^50,51^ License No. CC BY 3.0-DEED, 6082921251559, CC BY 4.0.

**Table 1:** Comparison between Bulk MOFs and 2D MOFs Based on their Specific Surface Area and Photocatalytic Capability

	Bulk MOFs					2D MOFs					
S. No.	nanocomposite	specific surface area	light response range	applicability	activity	nanocomposite	specific surface area	light response range	applicability	activity	reference
1	bulk Zn-MOF-NH_2_	23.7 m^2^ g^–1^	visible region	CO_2_ reduction	0.047 μmol/h	Zn-MOF-NH_2_	48.4 m^2^/g	visible region	CO_2_ reduction	0.28 μmol/h	^48^
2	bulk PMOF (Ti)	110.5 m^2^ g^–1^	visible light	oxidation of sulfides	37.6 mmol g^–1^ h^–1^	2D PMOF (Ti)	300.6 m^2^/g	visible region	oxidation of sulfides	75.2 mmol g^–1^ h^–1^	^52^
3	bulk MOF 2	157.78 m^2^ g^–1^	visible and IR active	CO_2_ conversion	276.89 μmol g^–1^	nMOF 2	346.99 m^2^ g^–1^	visible and IR active	CO_2_ conversion	658.74 μmol g^–1^	^53^
4	3D bulk In-TCPP MOF	111.6 cm^3^ g^–1^	UV–vis spectrum	H_2_ production	5.87 μmol g^–1^ h^–1^	In-TCPP NS	626.1 cm^3^ g^–1^	UV–vis spectrum	H_2_ production	67.97 μmol g^–1^ h^–1^	^54^
5	3D MOF-Re	1768 m^2^ g^–1^	UV–vis spectrum	CO_2_ reduction	TON of 0.55	2D MOF-Re	-	UV–vis spectrum	CO_2_ reduction	TON of 27.8	^55^
6	bulk Cu-MOF-NH_2_	52.8 m^2^ g^–1^	visible region	Cr^6+^ reduction	-	2D Cu-MOF-NH_2_	69.7 m^2^ g^–1^	UV–vis active	Cr^6+^ reduction	-	^56^
7	Zn-MOF bulk	295 m^2^ g^–1^	visible light active	CO_2_ reduction	59.9 cm^3^ g^–1^	Zn-MOF nanosheets	445 m^2^ g^–1^	visible light active	CO_2_ reduction	103.8 cm^3^ g^–1^	^57^

## 2 Fabrication Processes of 2D MOF-Derived Photocatalysts

In recent years, a wide range of synthesis techniques have been employed to fabricate 2D MOFs. Among the most commonly reported methods were hydrothermal and solvothermal synthesis, electrostatic self-assembly, and in situ self-assembly, alongside other emerging strategies.^58,59^ These diverse approaches were broadly categorized into two principal strategies: top-down and bottom-up, as shown in Figure 2. The schematic (pie chart) illustrates the proportional distribution of various synthesis strategies employed in the preparation of 2D MOF-based photocatalysts. Each category encompassed specific methodologies that offered distinct advantages in terms of structural control, crystallinity, and scalability and were discussed comprehensively in the literature about their mechanistic pathways and practical applicability.^60^ The key fabrication parameters for 2D MOFs are summarized in Table 2. This provides a comparative overview essential for optimizing the design and scalability of 2D MOF-derived materials.

**Figure 2:**
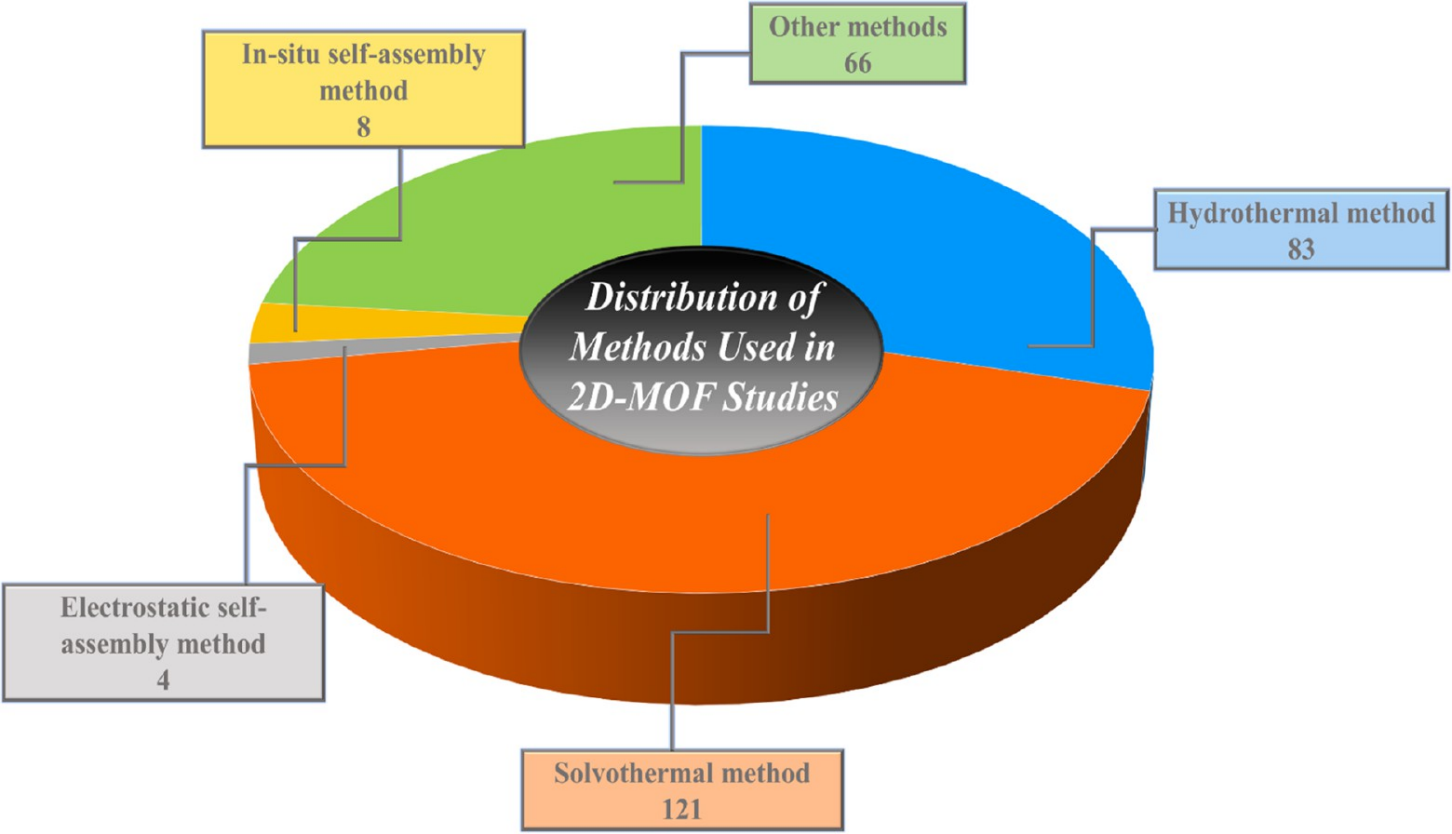
Schematic representation of the distribution of preparation strategies for 2D-MOF photocatalysts based on data retrieved from Scopus for the period 2019–2025, illustrated as a pie chart.

**Table 2:**
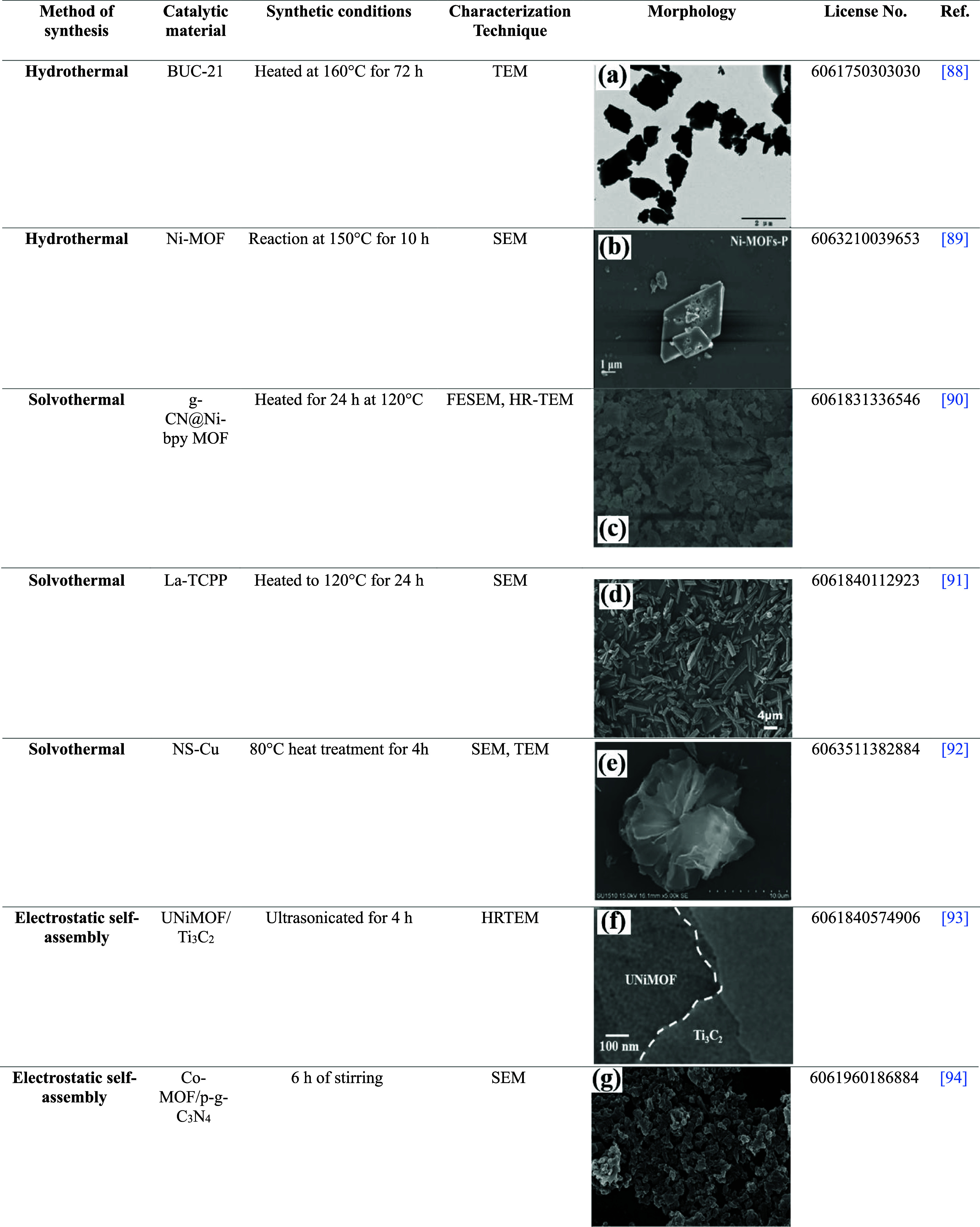

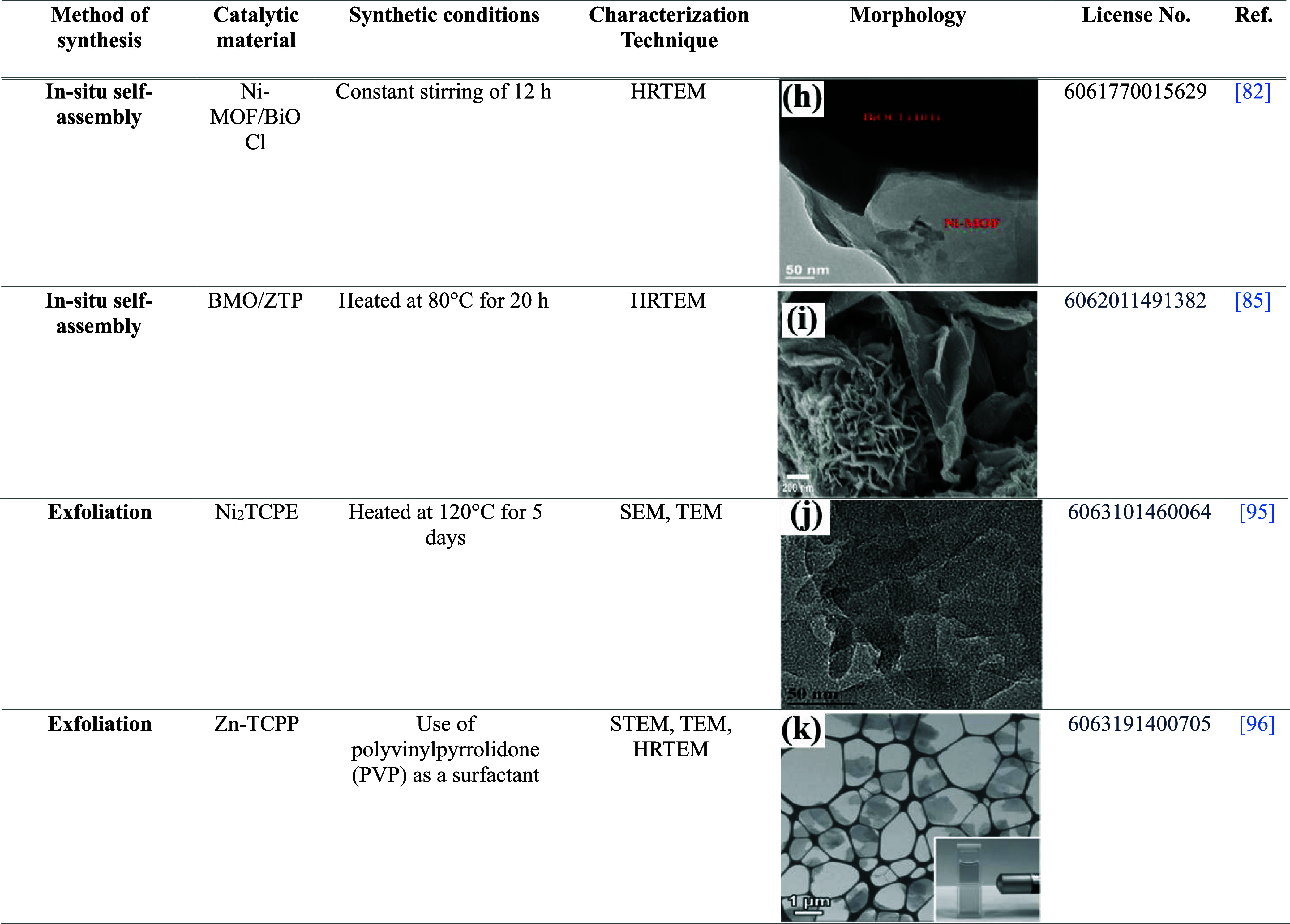
Summary of Reported Other 2D MOF-Derived Nanomaterials for Environmental Applications^88,89,92,93,95,96^

### 2.1 Hydrothermal Method

2D MOFs can be synthesized using the hydrothermal method (a bottom-up method) as it involves water as a solvent, reacting with reactants at a given temperature.^61,62^ For the most part, the synthesis of MOFs usually involves organic linkers such as hexa-substituted benzene^63,64^ and hexa-substituted triphenylene.^65,66^ These organic linkers are mostly used, but there are a few exceptions. As per the increasing demand for new MOFs, the main focus is on achieving precise control over the structural topology, including electronic structure, pore size, high porosity, and other such atomic properties. For example, Zheng and colleagues synthesized the hexahydroxytrinaphthylene (HHTN) ligand, partially soluble in water and capable of deprotonating catechol in the presence of base.^67^ The Cu_3_(HHTN)_2_ was prepared via a series of treatments, including mixing of HHTN with copper acetate in a mixed solution of DMI/H_2_O (3:1 v/v) and ammonia–water (25–28%), leading to the higher yield of 87%. The consistent structure of Cu­(HHTN)_2_ MOF was confirmed by powder X-ray diffraction, while TEM images revealed sheet-like morphology with multiple layers.

In another work, a successful S-scheme heterojunction MOF-BiOBr/Mn_0.2_Cd_0.8_S (MOF-BiOBr/MCS) was synthesized by Hua et al. via a hydrothermal in situ growth method.^68^ MOF–BiOBr/MCS nanocomposites were synthesized through a multistep process involving hydrothermal treatment. The mixed solution was sealed in an autoclave and heated for 6 h at 180 °C. Overall synthetic strategy was depicted schematically in Figure 3a, including three steps: (i) self-assembly of the Bi-MOF framework, (ii) thermal calcination to form MOF–BiOBr, and (iii) MCS nanoflower growth on the MOF–BiOBr surface due to hydrothermal treatment. Scanning electron microscopy (SEM) showed that pure BiOBr nanosheets aggregated significantly (Figure 3b), while MOF–BiOBr (Figure 3c) nanorods from the CAU-17 (Figure 3d) precursor reduced this agglomeration, enhancing catalytic potential. The 40% MOF–BiOBr/MCS composite exhibited a uniform distribution of cauliflower-like MCS structures (Figure 3e), improving the effective surface area and promoting heterojunction formation, indicating successful incorporation of MCS as a cocatalyst. Similarly, type-II 2D BiOIO_3_/rod-shaped Zn-MOF (BOIOZ-15) was prepared by Yuhong et al. through a hydrothermal approach.^69^ First, Zn-MOF and BiOIO_3_ were added to a solution of deionized water. They were then treated for 12 h at a higher temperature of 110 °C, after which washed with ethanol and deionized and dried. The morphology of BOIOZ-15, which has an attached lamellar BiOIO_3_ surface over rod-like Zn-MOF, was shown by SEM examination. Similarly, Xia et al. used the hydrothermal technique of synthesis to create a 2D Z-scheme heterojunction of MOF/In_2_S_3_ films (Al/InX %).^70^ The synthetic route involved the formation of In-doped Al-TCPP, which produced Al/In15% using CTAB and thioacetamide in deionized water, followed by a series of treatments such as heating at 95 °C along with 1.5 h of reflux. The prepared mixture was centrifuged and washed with deionized water and ethanol. The stacked nanomorphology with small particles was determined for the Al/In15% nanocomposite using SEM study.

**Figure 3:**
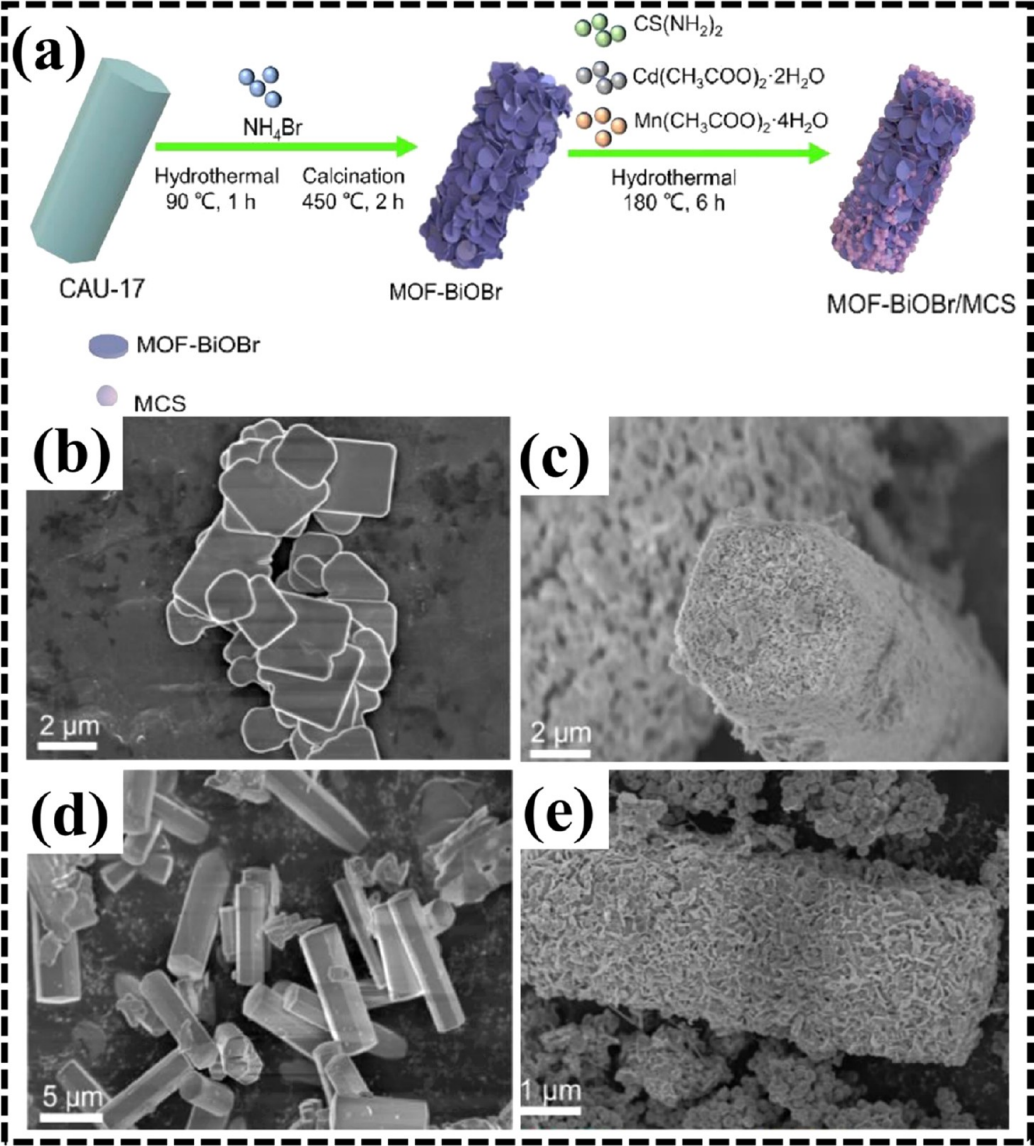
(a) Schematic representation of the fabrication procedure for the MOF–BiOBr/MCS nanocomposite, (b) SEM micrograph of BiOBr, (c) MOF-BiOBr, (d) CAU-17, and (e) 40% MOF–BiOBr/MCS composite revealed a uniform distribution of MCS nanoflowers. Redrawn with approval from Elsevier (license no. 6060721026370).^68^

### 2.2 Solvothermal Method

The solvothermal method involves different solvent ratios to synthesize 2D MOFs in a bottom-up approach.^71–73^ This method provides precise control over NP size, resulting in a high crystalline structure, and is characterized by the use of nonaqueous solvents such as ethanol, hydrochloric acid, methanol, etc.^74^ For instance, Jiang et al. synthesized NiTAA, forming four imine bonds coordinated with a Ni­(II) atom by a solvothermal procedure,^75^ using HATP·6HCl, Ni­(OAc)·4H_2_O, and 1,1,3,3-tetramethoxypropane dissolved in a mixture of solvents DMF, aqueous Et_3_N (triethylamine), and *n*-BuOH while maintaining the temperature of 120 °C for 5 days, resulting in the formation of NiTAA-MOF. The creation of the nanocomposite was confirmed via FTIR spectra. Also, using the solvothermal method, Nguyen and the team constructed a bismuth terephthalate (Bi-BDC) MOF composite.^76^ The specified amount of both H_2_BDC and Bi­(NO_3_)_3_·5H_2_O was dispersed in a solution of DMF followed by heat treatment of 120 °C in an oven using an autoclave. Strong peaks appeared at 1560.1 and 1381.7 cm^–1^ in the FTIR analysis, confirming that the carboxylate group of BDC and Bi^3+^ firmly coordinated. Moreover, SEM analysis expressed rounded spherical and rod-like morphology of Bi-BDC nanocomposite.

A one-pot solvothermal strategy was employed for the fabrication of a cobalt-based MOF material (CoBDC MOF) directly on layered Nb_2_CT_X_ MXene.^77^ Following the reaction, the product was dried at 60 °C for 12 h in an oven, yielding a composite material that served as the precursor for CoP@C and CoBDC-derived CoP/C@Nb_2_C catalysts. The overall synthetic route is illustrated in (Figure 4a), outlining the transformation from MOF to the final phosphide-based composite. FESEM images shown in (Figure 4b,c) confirmed that the layered architecture of the initial CoBDC@Nb_2_C composite was largely preserved after both calcination and phosphidation. However, the surface of the resulting CoP/C@Nb_2_C appeared smoother, and the underlying MXene sheets became more distinctly visible. Transmission electron microscopy (TEM) of the final composite (Figure 4d) revealed discrete CoP nanoparticles uniformly embedded within a carbonaceous matrix. High-resolution TEM (HRTEM) analysis confirmed the presence of well-defined CoP crystallites, with an interplanar spacing of 2.47 Å, corresponding to the (111) diffraction plane of orthorhombic CoP, thus verifying the successful formation of the desired phase. Similarly, Nguyen and colleagues prepared bismuth and trimesic acid-based MOF composite Bi-BTC DMF/MeOH via utilizing the solvothermal approach using a mixture of solvents DMF and MeOH.^78^ The procedure involved dispersion of H_3_BTC and Bi­(NO_3_)_3_·5H_2_O in a mixture of DMF and MeOH solution and constant stirring for half an hour, followed by heating treatment in an autoclave at 120 °C for 24 h. According to the results, the pure DMF environment was inactive for Bi-BTC production, while MeOH guaranteed that Bi-BTC formed effectively in the DMF environment. In contrast, the Bi-BTC-DMF variant displayed an irregular block-shaped structure, with particle sizes exceeding 500 nm (Figure 4d). The Bi-BTC-MeOH sample exhibited a distinct plate-like morphology with lateral dimensions below 5 μm, as observed in (Figure 4e). Notably, the Bi-BTC-DMF/MeOH composite presented a rod-like morphology, with widths ranging from approximately 200 to 300 nm, reflecting the influence of solvent composition on the crystal growth behavior and final particle shape.

**Figure 4:**
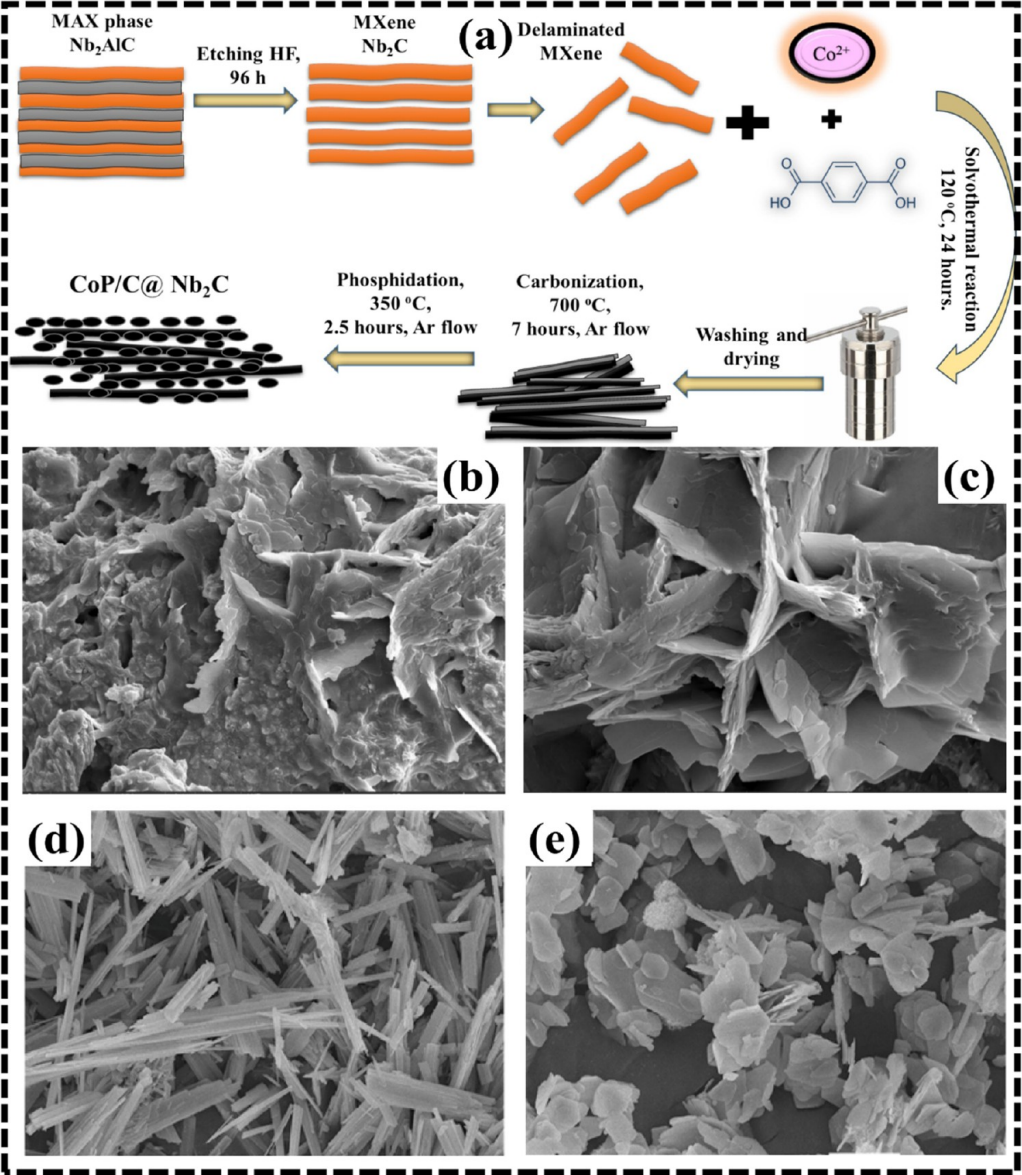
(a) Synthesis process of CoP/C@Nb_2_C. (b,c) FESEM micrographs. Reprinted with permission from Elsevier (license no. 6061191000821).^77^ (d,e) SEM micrographs of Bi-BTC-DM. Adapted with approval from Elsevier (license no. 6061200786097).^78^

### 2.3 Electrostatic Self-Assembly Method

To organize and assemble nanoparticles within a specific medium, this method leverages electrostatic interaction forces.^79,80^ Thus, the desired structure can be obtained through various arrangements and orientations of particles in an orderly manner by regulating the electrostatic force of interaction.^47^ Similarly, Jiang et al. successfully utilized the electrostatic method of self-assembly treatment and fabricated a 2D S-scheme heterojunction CABB/Ni-MOF nanocomposite.^81^ The nanocomposite was synthesized using an ultrasonicated solution of Ni-MOF in ethyl acetate, followed by the addition of CABB with 10 h of stirring, onward dried at 50 °C for 12 h. The morphological images obtained via TEM clearly showed that ultrathin Ni-MOF nanosheets were covered with a pile of 2D rectangular nanosheets of CABB. Furthermore, since all of the characteristic peaks for CABB and Ni-MOF were present, the XRD spectrum confirmed the presence of all the elements in the CABB/Ni-MOF.

In another work, using the electrostatic method of self-assembly, Zhou and the team fabricated a 2D nanocomposite Ni-MOF/BiOCl.^82^ The synthetic procedure involved mixing Ni-MOF and BiOCl nanosheets in a 20 mL IPA solution, followed by 12 h of stirring and overnight vacuum-drying at 60 °C. The electrostatic self-assembly strategy resulted in a Ni-MOF/BiOCl heterojunction with stacked nanosheets, as illustrated in Figure 5a. SEM (Figure 5b,c) and TEM analyses revealed the material’s morphology, with the Ni-MOF displaying ultrathin, flexible structures with micrometer-scale dimensions and BiOCl showing rectangular nanosheet morphology. TEM images confirmed that BiOCl sheets were anchored to the Ni-MOF nanosheets, forming a direct 2D/2D interface. Zhang et al. created 2D Z-scheme heterojunction g-C_3_N_4_/Ni-MOF (NMF/CN-9) using the electrostatic method of self-assembly.^83^ Ni-MOF and g-C_3_N_4_ were dispersed in ethanol solution to create the NMF/CN nanocomposite, which was then stirred for six hours and dried at 60 °C. XRD analysis revealed the nanocomposite’s preserved crystallinity, while the FTIR spectrum showed the effective creation of the NMF/CN photocatalyst via verifying the presence of functional groups.

**Figure 5:**
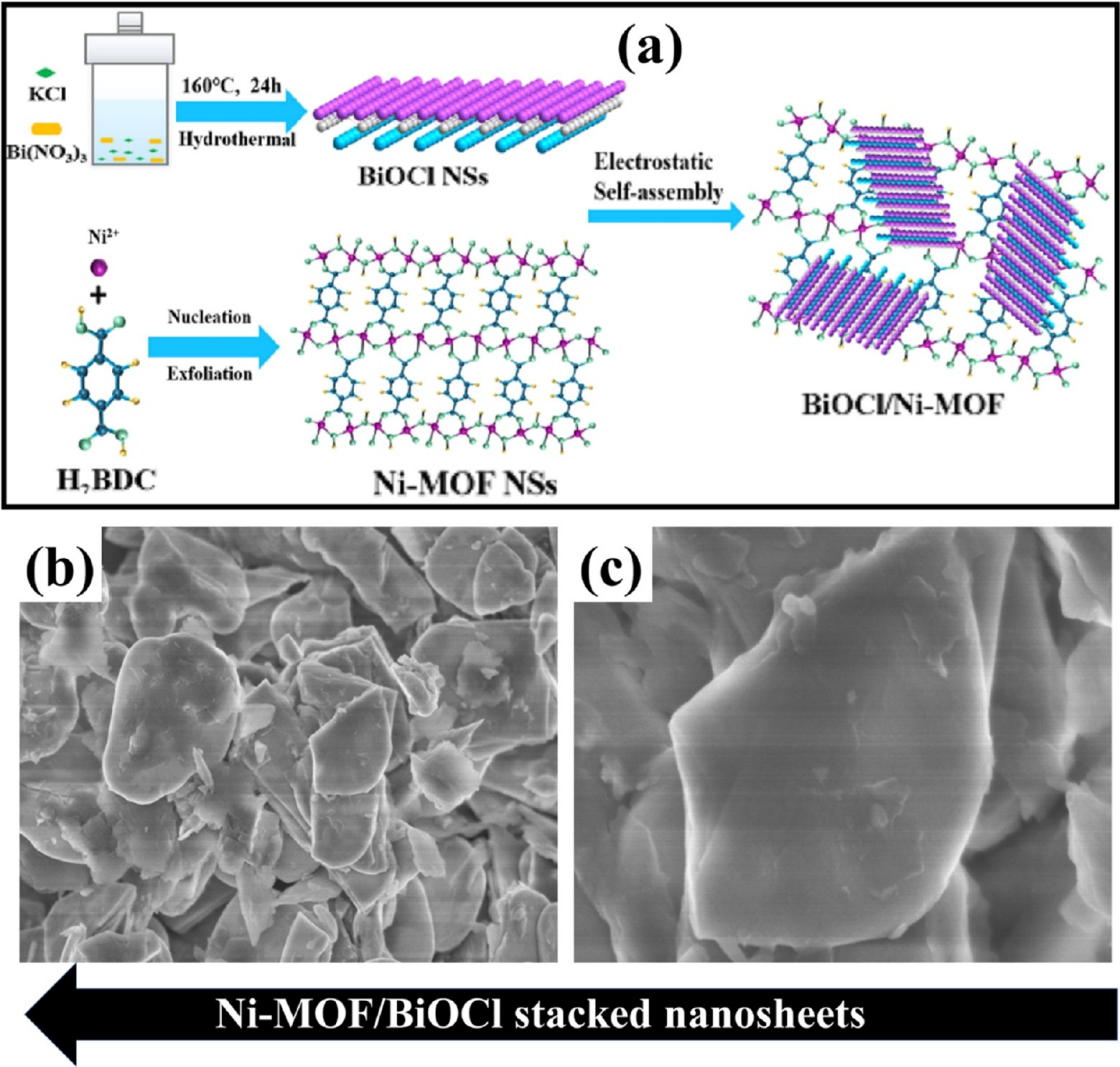
(a) Schematic illustration depicts the synthetic pathway of the Ni-MOF/BiOCl nanostructure. (b,c) SEM micrographs of Ni-MOF/BiOCl. Reproduced with permission from Elsevier (license no. 6061230348631).^82^

### 2.4 In Situ Self-Assembly Method

The in situ self-assembly method is a bottom-up approach where metal ions and organic ligands spontaneously organize into 2D MOF structures directly within the reaction medium. This method offers simple, scalable synthesis under mild conditions, often using surfactants or templates to control morphology and enhance material functionality.^84^ For example, a 2D/2D Bi_2_MoO_6_/Zn-TCPP (BMO/ZTP) heterojunction was synthesized through an in situ self-assembly growth strategy.^85^ The synthesis procedure, illustrated in Figure 6a, involved heating the precursor mixture at 80 °C for 20 h. Scanning electron microscopy (SEM) images (Figure 6b,c) revealed that Bi_2_MoO_6_ exhibited a nanosphere-like morphology composed of irregularly arranged nanosheets, consistent with its well-known 2D sheet-like structure. Notably, the SEM (Figure 6d) and TEM images of the BMO/ZTP-0.5 composite confirmed a closely stacked configuration between the two 2D materials. During the Zn-TCPP self-assembly process, Bi_2_MoO_6_ functioned as a nucleation substrate, directing the growth of Zn-TCPP nanosheets across its surface. This interaction, supported by the pleated morphology of Zn-TCPP, effectively encapsulated the Bi_2_MoO_6_ nanospheres, thereby enhancing interfacial contact and promoting the formation of a tightly integrated 2D/2D heterojunction. Transmission electron microscopy (TEM) analysis (Figure 6e,f) further validated the 2D nature of both Bi_2_MoO_6_ and Zn-TCPP components. The presence of extended lamellae and pleated nanosheet architecture was indicative of high surface area and large interfacial contact zones, critical factors for forming intimate heterojunctions. The HRTEM image (Figure 6g) revealed a well-defined interface between Bi_2_MoO_6_ and Zn-TCPP, with a distinct lattice fringe spacing of 0.326 nm, corresponding to the (131) plane of Bi_2_MoO_6_.

**Figure 6:**
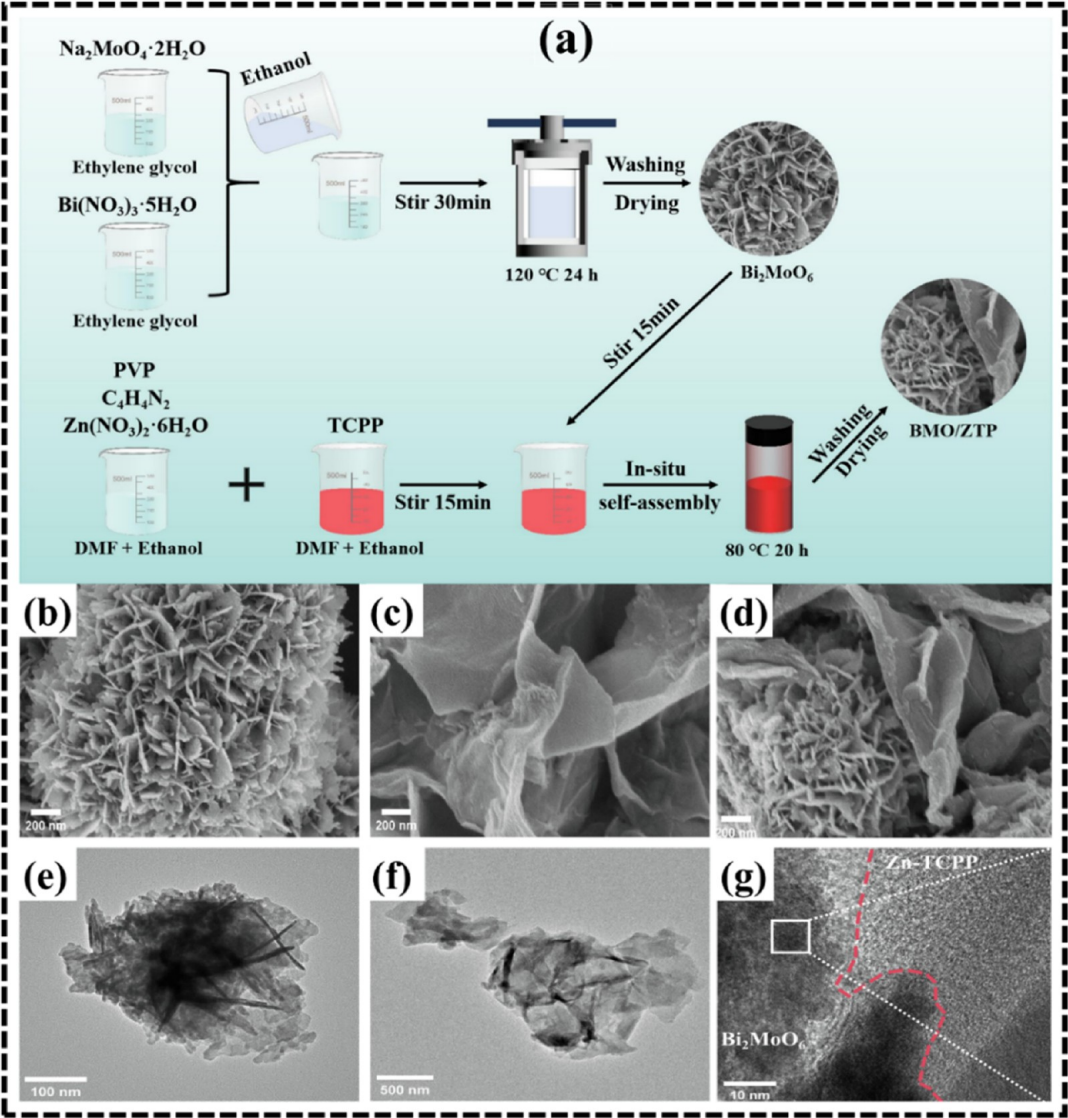
(a) Illustration of the synthetic route for the BMO/ZTP heterojunction, (b,d) SEM micrographs of Bi_2_MoO_6_/Zn-TCPP, (e,f) TEM micrographs, and (g) HRTEM micrograph. Redrawn with approval from Elsevier (license no. 6061291147779).^85^

### 2.5 Other Methods

The other synthetic strategies such as in situ precipitation and gentle heating can also be employed for the synthesis of two-dimensional MOF-based nanocomposites with good photocatalytic activity. For instance, a 2D/2D ZIF-L/g-C_3_N_4_ (ZCx) Z-scheme photocatalyst was created via an in situ precipitation approach and used to produce H_2_O_2_.^86^ In the synthetic process, g-C_3_N_4_ (50%, w/w) aqueous solution was combined with the precursors Zn­(NO_3_)_2_·6H_2_O and 2-methylimidazole, then stirred for 4 h. After centrifugation and water washing, the nanocomposite ZCx was produced by drying it in an oven set at 60 °C for 12 h. The SEM study of ZC50 revealed dispersed sheets of g-C_3_N_4_ over the leaf-like surface of ZIF-L. The study discovered that the 2D/2D ZIF-L/g-C_3_N_4_ heterostructure significantly inhibited g-C_3_N_4_ from agglomerating. In a different investigation, Zhao and colleagues used easy ultrasonification and mild heating to create a 2D Zn-MOF/BiVO4 S-scheme heterojunction.^87^ After adding Zn-MOF and BVON to an ethanol solution, they were ultrasonicated for 30 min and then heated for 4 h at 70 °C. The TEM image used to describe the nanocomposite xZn-MOF/BVON showed that BVON was attached to Zn-MOF nanosheets. Additionally, the XPS spectra showed that the heterojunction was successfully formed and that, in comparison to BVON, the 20Zn-MOF/BVON nanocomposite had a negative shift in Bi 4f and V 2p. Additionally, Zn 2p and N 1s showed a favorable shift for the nanocomposite compared to Zn-MOF, indicating a significant contact between Zn and –OH groups over BVON, which was responsible for the effective compounding in heterojunction formation.

The surface properties of 2D MOFs, such as porous design, exposed surface area, and modified surface groups, have a major influence on the performance of composites derived from it.^97,98^ Furthermore, a thorough comprehension of how various synthetic techniques affect surface phase characteristics offers helpful direction in the creation and composition of succeeding variants, allowing enhancement of functionality in particular applications. Ultrasonic exfoliation is one of the most often used top-down methods, which includes ease of usage.^97^ Long-term ultrasonication can damage large crystals, so techniques like chemical exfoliation and electrochemical etching are preferred to maintain the dimensional stability of 2D MOFs while improving nanosheet production. Hydrothermal and solvothermal methods are commonly used for synthesizing nanocomposites due to their tunable structure, ease of use, and capacity to produce superior 2D MOFs in mild environments.^74,97^ These methods have drawbacks, including the need for expensive autoclaves, longer reaction times, and challenges in real-time monitoring. Pyrolysis is an alternative synthesis approach that, alongside solvothermal and hydrothermal techniques, is comparable with other synthesis approaches.^99^ Carbon-based derivatives are created by removing organic ligands from 2D MOFs through thermal treatment in an inert gas. Despite advancements in the synthesis of 2D MOF, issues like customization, stability, multipurpose layout, and manageability still exist, hindering their broader applications.

## 3 Various Heterojunction Strategies of 2D Composite MOFs

Most MOF materials have a relatively wide band gap, promoting less efficient utilization of solar light. Additionally, their poor quantum yield and electron flow limit their photocatalytic activity.^100,101^ Thus, their photocatalytic performance or activity can be boosted by combining with a semiconducting material in such a viable and efficient way.^102,103^ Due to porous and larger surface area of 2D MOFs, the dispersion of the respective semiconductor becomes easier in this type of strategy of modification strategy of two-dimensional MOF material.^104^ This facilitates a homogeneous distribution of active sites and avoids aggregation.^105^ Moreover, heterojunctions can enhance the light absorption to some scale while reducing the reverse charge fusion and thus uplifting their migration proficiency.^106,107^ Various heterojunctions have been constructed by combining different semiconductor materials with 2D MOFs. At present, the main research directions to construct heterojunction based on 2D MOFs mainly include type-II, Z-scheme, and S-scheme heterojunctions.

### 3.1 Type-II Heterojunctions

Analogous to semiconductor band theory, type-I (staggered), type-II (straddling), and type-III (broken gap) are the three heterojunctions between semiconductors (SC) and hybrid frameworks.^108^ Out of these, type-II heterojunctions have higher LUMO as well as HOMO energies when compared with CB and VB of SC, respectively.^109,110^ And this type of arrangement prevents the carrier recombination as it promotes the e^–^ transfer to 2D MOF from SC while transfer of h^+^ from 2D MOF material to SC.^69^ In a study, Niu and the team synthesized a type-II 2D/2D heterojunction BiOIO_3_/Bi-MOF (BOIOB-*x*) via the solvothermal method.^111^ The prepared BOIOB-*x* showed good optical capability with smaller absorption fringes when compared to BOIO regardless of the loaded mass of Bi-MOF nanomaterial. However, an increase in BOIOB-*x*’s absorption level enhances its capacity to absorb light. As obtained in PL study, the effective compounding of Bi-MOF in the heterostructure resulted in lower PL intensity for the BOIOB-*x* (BOIOB-30) composite, indicating effective separation of photocarriers. In transient photocurrent experiments and EIS, the BOIOB-30 nanocomposite showed higher photocurrent density and lower arc radius, indicating improved charge separation and transfer capabilities. It was proposed that in Bi-MOF/BiOIO_3_ composites, photogenerated electrons move from the VB to CB of BiOIO_3_ and Bi-MOF, respectively, as the CB potential of Bi-MOF is inferior to BiOIO_3_. This allows the direct conversion of CO_2_ into CO, aided via transfer of photoexcited holes to BiOIO_3_, thereby enhancing the uncoupling of e^–^ and h^+^ in the BOIOB-30 nanocomposite.

In another example, Yuhong and team successfully fabricated a 2D BiOIO_3_/Zn-MOF type-II heterojunction using BiOIO_3_ and Zn-MOF.^69^ A photoluminescence (PL) study on the BiOIO_3_/Zn-MOF nanocomposite revealed a reduced PL peak intensity, indicating lower recombination rates of photogenerated charge carriers. Electrochemical impedance spectroscopy (EIS) showed a smaller arc radius for the nanocomposite, suggesting lower charge transfer resistance and enhanced photocatalytic activity. The transient photocurrent study indicated a higher photocurrent density due to effective photocarrier separation. The photocatalytic mechanism was analyzed using UV–visible spectroscopy and Mott–Schottky plots, revealing e^–^ excitation from VB to CB of both composites, facilitating charge migration. Electrons move from the CB of Zn-MOF to BiOIO_3_, while holes move in the opposite direction, contributing to photocatalytic activity. The Type II heterojunction promotes charge separation but faces challenges such as hindered carrier migration at the interface and lower redox potentials. Further modifications are needed to overcome these limitations. Li and colleagues produced and used a 2D/2D type-II heterojunction Co-TCPP MOF@B-TiO_2–*X*_ (BTC-*y*) for BPA photodegradation using a hydrothermal approach.^112^ PL, EIS, and transient photocurrent response were among the investigations that demonstrated the nanocomposite’s superior charge separation, transfer, and migration efficiency. Additionally, Mott–Schottky (M–S) plots showed that Co-TCPP MOF and B-TiO_2–*X*_ materials expressed n-type behavior. The research, as mentioned earlier, demonstrated that the nanocomposite’s (BTC-10%) improved charge separation efficiency was caused by an added oxygen vacancy, which also widened the light response range and inhibited charge recombination. Furthermore, light utilization capability of the nanocomposite along with Co-TCPP MOF and B-TiO_2–*X*_ is illustrated in Figure 7a using UV–vis DRS study, while Figure 7b displayed a decrease in band gap energy of the nanomaterial, resulting in enhanced light response range. The photocatalytic activity is shown in Figure 7c following a trend of BTC-10% (96%) > Co-TCPP MOF (50%) > B-TiO_2–*X*_ (20.7%). Additionally, a k value of 7.3 times higher was recorded in the as-prepared photocatalyst than in pure Co-TCPP MOF and 19.3 times higher than in B-TiO_2–*X*_ nanomaterial (Figure 7d). The type-II charge migration pathway was illustrated, with Co-TCPP MOF showing a larger LUMO compared to B-TiO_2–*X*_. Moreover, photogenerated e^–^s migrated toward CB of B-TiO_2–*X*_ from the CB of Co-TCPP MOF when exposed to sunlight, whereas holes migrated toward Co-TCPP MOF. The lower VB energy of Co-TCPP MOF prevented production of ^•^OH radicals, but it was able to make ^•^O_2_^–^ radicals since CB energy level of B-TiO_2–*X*_ was greater than that of O_2_/^•^O_2_^–^ (−0.33 eV versus NHE) (Figure 7e).

**Figure 7:**
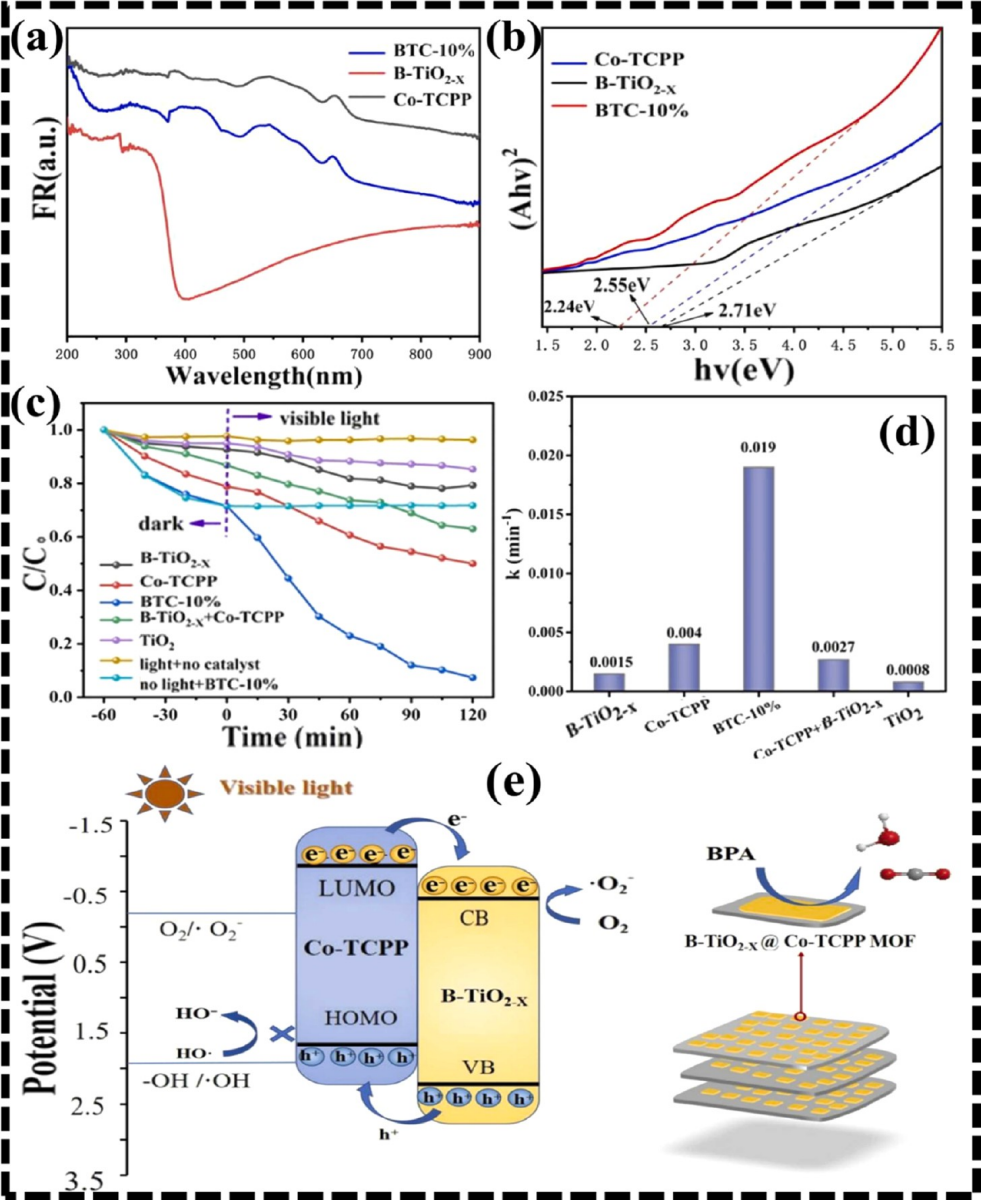
(a) UV-DRS, (b) Tauc plots, (c) photocatalytic performance, (d) corresponding kinetic rates of fabricated photocatalysts, and (e) proposed photocatalytic mechanism of BTC-10% under visible light. Adapted with approval from Elsevier (license no. 6071800734044).^112^

### 3.2 Z-Scheme Heterojunction

Z-scheme heterojunctions enhance interfacial charge separation by reducing electron recombination losses and broadening spectral response. This significantly improves catalytic performance, making them more advantageous than type-II heterojunctions.^113^ Although type-II heterojunction helps in increasing charge separation efficiency, it compromises the redox potential of the nanocomposite, thus providing an undesirable photocatalytic activity or performance.^114^ In a Z-scheme heterojunction, electrons transfer from semiconductor A (higher Fermi level) to semiconductor B (lower Fermi level) until equilibrium is reached. This process generates an internal electric field at the junction, directed from A to B.^113,115^ Simultaneously, the energy bands of semiconductors A and B bend in opposite directions, with A bending upward and B bending downward. When exposed to light radiation, the resulting photocarriers recombine at the contact, promoting the recombination of electrons and holes with notable reducing and oxidizing properties in the heterojunction. Z-scheme heterojunctions are classified into direct (DZS) and indirect (IZS) types depending on the e^–^ migration mediators.^116^ Out of which, all-solid-state and liquid-phase are two types of IZS on the basis of e^–^ localize medium present.^117,118^ The liquid-phase Z-scheme heterojunction combines two different SC with e^–^ acceptor and donor pairs. Upon photoirradiation, electrons localize toward greater reduction potential (RP), while h^+^ remain at the side with a greater oxidation potential (OP), ensuring high redox capability. Conversely, the all-solid-state Z-scheme heterojunction utilizes metal nanoparticles (NPs) as e^–^ mediators, addressing reverse reaction issues present in liquid-phase systems. This configuration enhances redox capability and charge separation, outperforming previous creations and establishing greater efficiency in DZS heterojunctions.^119^

In a study of g-C_3_N_4_/Ni-MOF heterostructures, an induced electric field (EF) was observed at the contact, oriented toward Ni-MOF from g-C_3_N_4_, due to charge accumulation.^120^ Upon light irradiation, both materials experienced photoexcitation, generating e^–^–h^+^ pairs. The EF and band bending facilitated electron transfer from Ni-MOF to g-C_3_N_4_, allowing reassembly of e^–^s with h^+^. This created a DZS pathway, enhancing redox potentials at both catalysts’ surfaces (Figure 8a). To support the charge migration mechanism, density functional theory (DFT) analysis evaluated work functions (W) of g-C_3_N_4_ (002 plane) at 4.02 eV and Ni-MOF (100 plane) at 6.07 eV. The Fermi energy (FE) of g-C_3_N_4_ (−2.47 eV) was greater than Ni-MOF (−3.23 eV) (Figure 8b,c). This intrinsic potential difference drove electron transfer from g-C_3_N_4_ to Ni-MOF till the Fermi level equilibrium was reached. Photoluminescence (PL) studies provided additional evidence of this interfacial charge behavior. As depicted in Figure 8d, the Ni-MOF/g-C_3_N_4_ composites (1–4 ratio) demonstrated the lowest PL intensity compared to individual components and simple physical mixtures, indicating superior charge separation and reduced recombination. Band gap estimations derived from UV–Vis spectra confirmed that Ni-MOF and g-C_3_N_4_ exhibited optical band gaps of 3.0 and 2.68 eV, respectively (Figure 8e). The proposed PODS mechanism, illustrated in Figure 8f, explained the process in detail. Both 2D/2D Ni-MOF and g-C_3_N_4_ produced e^–^–h^+^ pairs in the presence of light. In the heterostructure, g-C_3_N_4_ had a lower conduction band potential (−0.92 eV vs NHE) compared to Ni-MOF (−0.59 eV vs NHE); however, Ni-MOF exhibited a higher valence band potential (2.41 eV vs NHE) relative to g-C_3_N_4_ (1.76 eV vs NHE). These energetic alignments effectively supported the creation of the ZS charge transportation pathway, maximizing charge carrier utilization for photocatalytic reactions. Similarly, Zhou et al. fabricated a 2D Cu-FeTCPP MOF/ZnTi-LDH hybrid nanocomposite using the surfactant-assisted method utilized for the degradation of CO_2_.^121^ Various studies were conducted to evaluate the charge isolation and migration in photocatalytic activity. The PL spectrum showed a low-intensity peak for the Cu-FeTCPP MOF/ZnTi-LDH hybrid composite, indicating effective charge carrier separation and a lower recombination rate. TRPL analysis revealed a longer fluorescence lifetime linked to improved photocatalytic activity. The photocurrent response demonstrated a higher curve value for the nanocomposite, indicating better charge transfer. The direct Z-scheme mechanism involved electron excitation from the VB to CB of both ZnTi-LDH and Cu-FeTCPP MOF under light, leading to recombination and retention of charge carriers, enhancing redox capability. This facilitated the reduction of CO_2_ into CO through strong reducible electrons, while holes generated OH· radicals from water.

**Figure 8:**
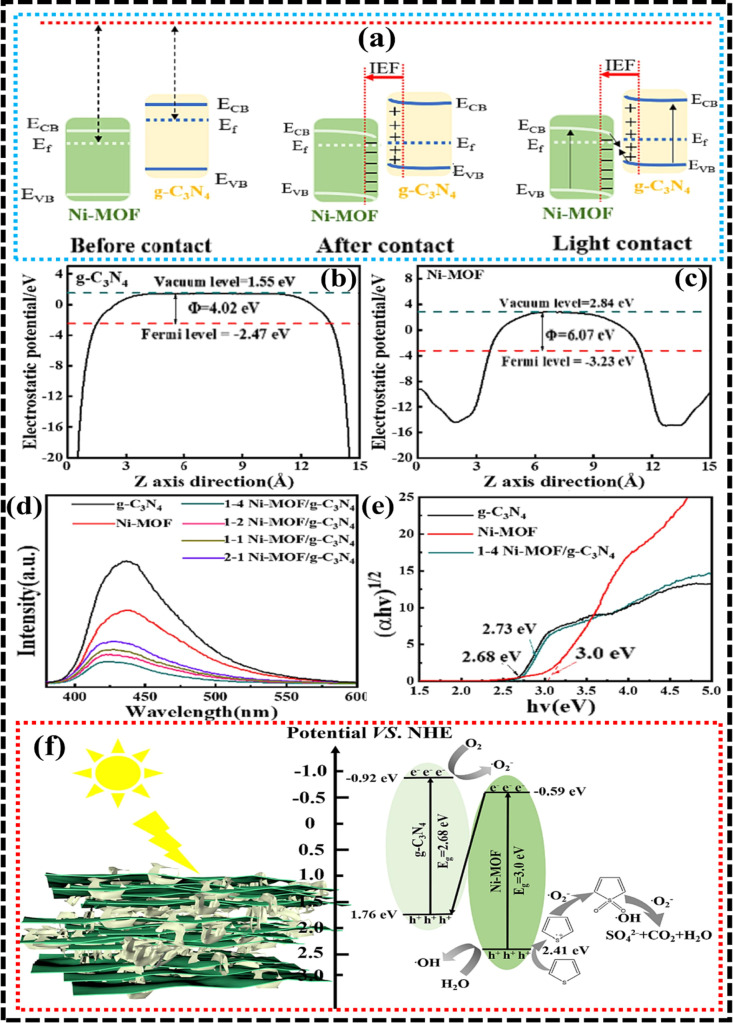
(a) Schematic diagrams illustrating the Fermi level alignment and energy band bending between g-C_3_N_4_ and Ni-MOF, highlighting the charge redistribution at the heterojunction interface, (b,c) work function measurements of g-C_3_N_4_ and Ni-MOF, respectively, (d) photoluminescence (PL) spectra, (e) estimated band gaps of g-C_3_N_4_, Ni-MOF, and the Ni-MOF/g-C_3_N_4_ heterostructure, and (f) proposed photocatalytic desulfurization mechanism for the Ni-MOF/g-C_3_N_4_ composite under visible light irradiation. Reproduced with permission from Elsevier (license no. 6074160692816).^120^

Additionally, Meng and colleagues created a 2D/2D Z-scheme heterojunction ZIF-L/g-C_3_N_4_ (ZCx) at room temperature using the in situ precipitation approach.^86^ The nanocomposite’s 2D/2D heterostructure successfully inhibited the g-C_3_N_4_ agglomerated activity, and an EDS analysis showed that Zn, C, N, and O were present and distributed throughout the nanocomposite. PL, TRPL, and EIS were among the tests used to assess the ZCx nanocomposite’s photocatalytic performance through charge segregation and shifting efficacy. Furthermore, directional decoupling of charge carriers via Z-scheme charge migration configuration and the dual-channel route was shown via DFT analysis. ZCx demonstrated enhanced photon harvesting characteristics by absorbing light, which was a characteristic shared by ZIF-L and g-C_3_N_4_ determined by UV–vis DRS spectra. Undesired charge separation and reverse reactions occur in MOF-based liquid-phase heterojunctions when e^–^s migrate toward MOFs’ CB from CB of SC.^114,122^ Besides, the use of noble metals as e^–^ intermediary in MOF-based all-solid-state Z-scheme heterojunction makes it expensive. Also, the light absorption capability of the semiconductor is impeded by metal NPs, along with their controversial charge transfer mechanism.^122,123^ The direct Z-scheme photocatalyst is more cost-effective, maintains a high redox capability than previous creations, relies on the direct contact of two semiconductors and thus does not require any electron mediator. This design is analogous to the S-scheme heterojunction. The DZS heterojunction is often mistakenly perceived to have same limitations as those of antecedent outmoded generations of Z-scheme heterojunctions. As a result, the concept of the S-scheme heterojunction was proposed.^124,125^

### 3.3 S-Scheme-Based Heterojunction

The drawbacks of previously discussed strategies of synthesizing heterojunctions are resolved with the introduction of S-scheme-based heterojunctions.^41^ This heterojunction, comprising two n-type semiconductors with an ‘S’ shaped pathway of shifting photogenerated carriers having high redox capacity, was presented by Yu et al. in 2019.^126–128^ OP exhibits more +ve VB energy and lesser *E*_f_ compared to RP. The reduction and oxidation photocatalysts are connected, leading to electron accumulation at the CB of OP from RP. This creates a varying charge density at their interface, inducing an electric field and causing RP’s band structure to bend upward while OP’s bends downward.^127,129^ The induced electric field (IEF) and Coulomb interplay facilitate pairing of electrons and holes in OP and RP, while inhibiting reverse reactions and preventing the movement of electrons from RP to OP. This promotes effective charge transfer and separation, maintaining structural simplicity. Compared to previous heterojunction strategies, S-scheme heterojunctions are easier to implement.

For example, Zhou and the team developed a 2D/2D Ni-MOF/BiOCl heterojunction using a self-assembly method as a synthetic route.^82^ The photocatalytic mechanism was demonstrated (Figure 9a), revealing excitation of Ni-MOF and BiOCl under the influence of light radiation and resulting in the formation of photogenerated e^–^ and h^+^. An electron migrated from CB of BiOCl to CB of Ni-MOF as *E*_f_ of BiOCl is greater, thus introducing IEF. Under the action of IEF, recombination occurred as photogenerated e^–^ migrated from Ni-MOF to interact with h^+^ on VB of BiOCl. Thus, e^–^ and h^+^ with higher reductive and oxidative powers were accumulated on CB of BiOCl and VB of Ni-MOF, respectively. These e^–^–h^+^ pairs react with absorbed O_2_ and surface water, bringing redox reactions that cause the creation of ^•^O_2_^–^ and OH^•^ radicals which on interaction with pollutant molecules lead to their degradation. Studies such as EIS and transient photocurrent were explored to understand charge transfer efficiency and separation (Figure 9b,c). EIS spectra revealed a semicircle radius of the Nyquist plot for the Ni-MOF/BiOCl nanocomposite, indicating a lower value of resistance toward charge separation. Additionally, higher value of photocurrent signals for the as-prepared photocatalyst was obtained in the transient photocurrent study, thus indicating a higher efficiency of charge transfer and separation. Similarly, a straightforward ultrasonication approach was used to generate 2D S-scheme heterojunction Zn-MOF/BVON.^87^ The XPS spectra of the prepared nanocomposite showed a negative shift in the binding energies of V 2p and Bi 4f, while Zn 2p showed a positive shift when compared to BVON and Zn-MOF. Using Tauc plots created from UV–vis absorption spectra, the corresponding band gaps of Zn-MOF and BVON were discovered to be 1.89 and 2.34 eV, respectively. The studies revealed n-type nature of BVON and Zn-MOF, with their CB levels at −0.32 V and −0.76 V vs NHE, respectively (Figure 9d). After forming the heterojunction, electrons transferred to BVON from Zn-MOF, creating IEF and causing upward band bending in BVON and downward bending in Zn-MOF. The S-scheme heterojunction boosts charge isolation while preserving the superior redox capability of materials, though its practical applications remain relatively unexplored.^114^

**Figure 9:**
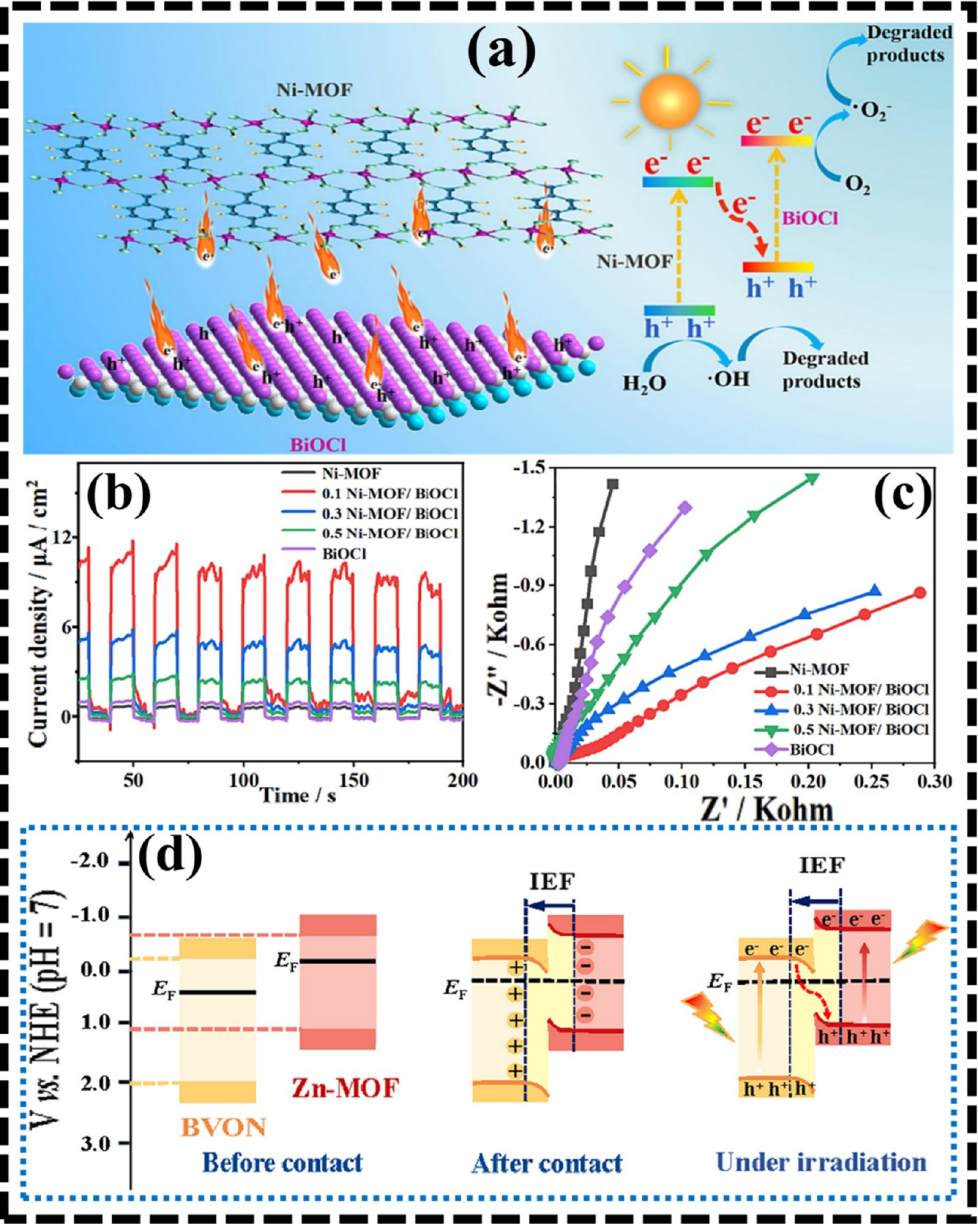
(a) The charge transfer mechanism and photocatalytic degradation of TC over the Ni-MOF/BiOCl heterojunction. Transient photocurrent (b) and EIS spectra (c) confirmed enhanced charge separation in the Ni-MOF/BiOCl system compared to individual components. Redrawn with approval from Elsevier (license no. 6074621060118).^82^ (d) Schematic depicts the formation of an S-scheme Zn-MOF/BVON heterojunction with an internal electric field promoting efficient charge transfer under visible-light irradiation. Reprinted with permission from Elsevier (license no. 6074630044483).^87^

Liu and group used the solvothermal approach to synthesize a 2D S-scheme heterojunction of ultrathin CdS nanosheets and Ni-MOF (NC*x*, where *x* = mass of CdS).^130^ The UV–vis DRS analysis revealed a substantial red shift in the generated nanocomposite with the addition of CdS along with the LMCT influence of Ni-MOF linked to an increased visible-light utilization range. The W of Ni-MOF and CdS were determined and revealed a lower Fermi level (*E*_f_) of Ni-MOF (−5.05 eV) than *E*_f_ of CdS (−4.47 eV). Therefore, electrons from CdS migrated toward Ni-MOF to equilibrate *E*_f_. This was confirmed by DFT analysis, indicating an e– migration of 0.72 e toward Ni-MOF through CdS, which resulted in the band bending due to the induced electric field within the nanocomposite. This band bending during light exposure encouraged e^–^–h^+^ recombination and tended to decrease fluorescence lifespan; however, inhibition of hole recombination in CdS resulted in an extension of lifetime. Thus, the relationship between enhanced redox capacity and S-scheme interfacial charge transfer was shown by UV–vis DRS and DFT experiments. In another example, an MOF-BiOCl/MoS_2_ photocatalyst was fabricated using a simple solvothermal method by Liu and group.^131^ Using various studies such as PL, EIS, and transient photocurrent responses, the uncoupling and migration efficiency of e^–^ and h^+^ were determined. The PL spectrum revealed lower PL intensity for the MOF-BiOCl/MoS_2_ nanocomposite, demonstrating lower recombination processes and efficient charge separation. The EIS study showed that a smaller arc radius led to reduced charge transfer resistance, while a superior photocurrent response was observed in transient photocurrent analysis. In the S-scheme charge migration mechanism of the MOF-BiOCl/MoS_2_ nanocomposite, light exposure generates e^–^ in CB while h^+^ in VB of BiOCl and MoS_2_. The recombination of photocarriers due to band bending allows for electrons with strong reducing 7K//capability at CB of MoS_2_ and holes with higher oxidizing capability at VB of BiOCl, facilitating the construction of reactive oxygen species (ROS).

## 4 Application of 2D MOF-Derived Photocatalysts

Water is essential for the survival of organisms, yet only 1% of the Earth’s water is drinkable, despite water covering 75% of the planet’s surface.^43^ This scarcity of potable water is largely due to rapid population growth, urbanization, and industrial activities that contaminate water supplies with pollutants such as heavy metals, organic dyes, pesticides, and antibiotics. For instance, the tanning industry produces one ton of leather, resulting in approximately 60 tons of wastewater loaded with heavy metals.^132,133^ Recent statistics highlight the annual production of about 700,000 tons of synthetic dyes, with around 200,000 tons released into the environment, showcasing the persistent and harmful effects of these contaminants.^134^ Furthermore, the overuse of pesticides and antibiotics results in residues that accumulate in soil, agricultural runoff, and the food supply, which can be significantly harmful.^135,136^ About 30% of antibiotic dosages are effectively absorbed, leaving the rest to be expelled into the environment. These toxic contaminants, often carcinogenic, accumulate in ecosystems, bioaccumulating through the food chain and posing long-term health risks to humans. According to the UN water summary status report for 2021, more than 200 million people reside in water-scarce regions and are compelled to consume tainted water, which causes almost one million fatalities from diarrheal illnesses each year.^137^ Additionally, the study mentions more than 251.4 million instances of schistosomiasis, a dangerous disease connected to contaminated water as reusing polluted water has become the only practical solution due to the depletion of clean water supplies. Thus, improvements in wastewater purification techniques are needed to tackle these significant health risks posed to consumers due to persistent drug residues in food chain.^138^ Throughout the last many decades, numerous practical applications of 2D MOFs have been studied, such as hydrogen production, CO_2_ conversion, organic synthesis, and organic pollutants removal.^39,139^ 2D MOF photocatalysts effectively respond to visible light and enhance catalytic performance through improved separation and mobility of photogenerated charge carriers. Their increased surface area and optimal pore size facilitate better pollutant adsorption, promoting simultaneous photodegradation or conversion into harmless environmental compounds.^140^ Composites derived from MOFs have been extensively studied for their potential in photodegrading organic pollutants such as textile dyes, pharmaceutical compounds, phenolic compounds, and antibiotics.^141–144^ The greater adsorption of pollutant molecules is due to the exceptional area of contact with tunable pore size of the MOF composite. The degradation energy required by pollutant molecules are specific to each molecule, aided by easy customization of the energy band gaps in MOF-based composites.^145,146^ Key aspects such as synthetic strategies, surface area, light harvesting region, photocatalytic applications, pollutant targets, efficiency, and stability assessments are systematically compiled for comparison.

### 4.1 Heavy Metal Removal

Heavy metals such as Cu, Fe, Hg, Pb and Cd are essential for physiological functions, but their excess poses significant toxicity risks to human health, and these constitute a class of metals with densities more than 4.5 g/cm3.^147–149^ Since they are resistant to biodegradation and experience significant bioaccumulation through untreated sewage discharge from anthropogenic activities, their longevity in the environment is worrying. There are serious concerns associated with the release of toxic ions in nature not only for humans but also for other living things.^150,151^ Thus, to eliminate this rising threat, MOFs with numerous applications have been proven as effective eliminators of these toxic ions, including Hg^2+^, Pb^2+^, along with oxyanions such as CrO_4_^2–^/CrO_7_^2–^, SeO_3_^2–^/SeO_4_^2–^, etc.^133^

For instance, a 2D water-stable nanocomposite MOF Zn­(Bim)­(OAc) developed using a facile method, having an ultrathin structure with exposed active sites, showed great affinity for heavy metals present in the waterbody, such as Pb.^152^ In earlier studies, XPS analysis of pristine Zn­(Bim)­(OAc)-NS revealed Zn, N, O, and C as primary constituents in the material. Following adsorption of Pb­(II) and Cu­(II), the appearance of distinct Pb 4f and Cu 2p peaks confirmed the successful binding of these toxic ions onto the Zn­(Bim)­(OAc)-NS surface. When tested individually, Zn­(Bim)­(OAc)-NS demonstrated high removal efficiencies for Cu­(II) and Pb­(II), reaching 98.2 and 99.1%, respectively, whereas Ni­(II), Co­(II), and Cd­(II) removal remained significantly lower at 12.3%, 11.6%, and 2.7%. This selectivity was further validated in mixed-metal systems having different heavy metals at 10 mg/L, which reduced the elimination efficiencies of Cu­(II) and Pb­(II) to 78.5 and 89.3%, with Cu­(II) experiencing a more pronounced inhibition effect. The prepared photocatalyst was designed for the rapid and effective removal of these metal ions, with an unleashed maximum adsorption capability of 253.8 mg/g for Pb­(II). This superior adsorption and higher selectivity for these metal ions over others resulted in the rapid completion of the saturation process within 90 min for Pb­(II).

In an investigation, 2D Ni-MOF nanosheets were prepared using a facile solvothermal method and employed for the synthesis of a Schottky junction Ni-MOF/NiB with enhanced photocatalytic capability.^153^ UV–vis diffuse reflectance spectroscopy of pure Ni-MOF showed a light absorption peak in the 300–550 nm range, attributed to ligand-to-metal charge transfer (LMCT) from O 2p to Ni^2+^ 3d orbitals, with an additional band at 600–800 nm for spin-permitted transitions (Figure 10a). Band gap energies decreased from 3.07 eV for Ni-MOF to 3.00, 2.90, and 2.85 eV for Ni-MOF/NiB-0.8, Ni-MOF/NiB-1, and Ni-MOF/NiB-1.2, respectively (Figure 10b). Under visible light, Ni-MOF/NiB-1 generated electron–hole pairs, promoting charge separation, while the NiB component acted as a cocatalyst, enhancing Cr­(VI) adsorption and photoreduction (Figure 10c). The photocatalyst was 96% efficient in the photoreduction of Cr­(VI) when exposed to sunlight for 75 min at pH of 2. However, an increase in the pH value displayed a negative impact with a performance drop from 96% to 20.6% of Ni-MOF/NiB-1 for Cr­(VI) reduction. Moreover, Ni-MOF/NiB exhibited superior reusability of up to three continuous cycles with very little loss in photocatalytic performance. Through simple reactant stirring, Wang and associates created 2D Cu-MOF-NH2 and 2D Fe/Cu-MOF-NH_2_, which were then effectively used for the photoreduction of Cr^6+^.^56^ It was shown that Fe/Cu-MOF-NH_2_ destroyed Cr^6+^ twice as efficiently as Cu-MOF-NH_2_. Additionally, the as-synthesized Fe/Cu-MOF-NH_2_ showed good reusability with no noticeable reduction in catalytic efficacy even after three cycles. DFT analysis demonstrated a notable ascent in the d-band center of 2D Fe/Cu (1:2)-MOF-NH_2_ to −2.52 eV, compared to −3.89 eV for pristine Cu-MOF-NH_2_ (Figure 10d). This shift indicated a lower energy barrier for e^–^ transfer to Cr­(VI) species by Fe sites. Additionally, charge-density difference analyses (Figure 8B,C) revealed significant charge localization over the interface in both Cu-MOF-NH_2_/Cr­(VI) and Fe/Cu (1:2)-MOF-NH_2_/Cr­(VI) complexes. Furthermore, Bader charge analysis showed Δ*Q* values of 0.55 and 0.87 e, respectively, with charge accumulation and depletion visualized in yellow and blue regions (Figure 10e).

**Figure 10:**
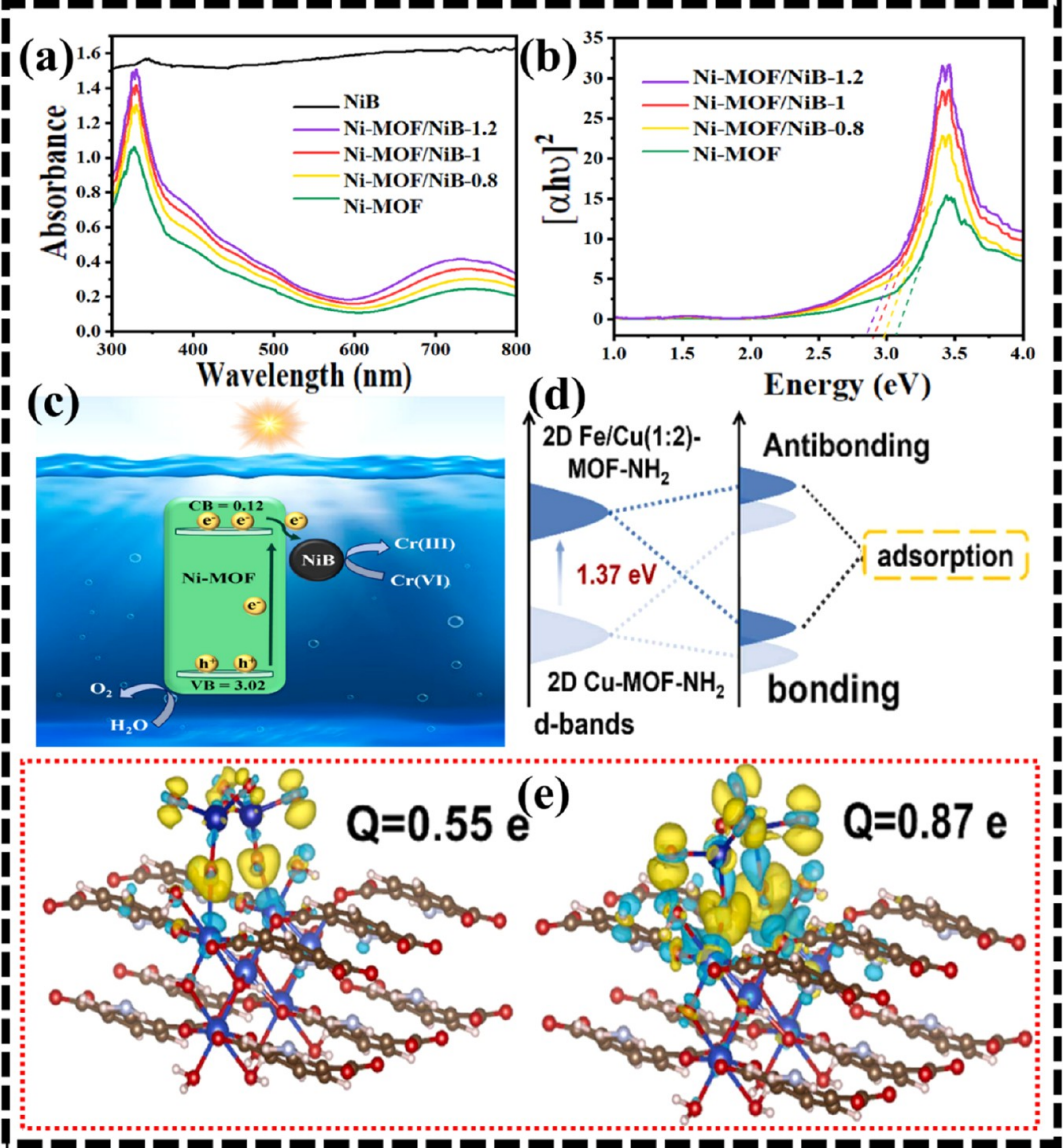
(a) UV–vis DRS spectra were recorded, (b) Tauc plots were plotted for Ni-MOF, Ni-MOF/NiB-0.8, Ni-MOF/NiB-1, Ni-MOF/NiB-1.2, and NiB, and (c) a mechanism for Cr­(VI) photoreduction over Ni-MOF/NiB was proposed. Adapted with approval from Elsevier (license no. 6075190599283).^153^ (d) Orbital interactions underlying catalyst–substrate binding were schematically illustrated and (e) charge-density difference images for 2D Cu-MOF-NH_2_ and Fe/Cu (1:2)-MOF-NH_2_ were visualized. Reprinted with approval from Elsevier (license no. 6075190846587).^56^

### 4.2 Dye Degradation

Wastewater frequently contains a diverse array of organic pollutants, surpassing inorganic contaminants in both variety and complexity.^154,155^ Among these, organic dyes, priority environmental pollutants, and pharmaceutical and personal care products (PPCPs) stand out as particularly concerning. Due to the elevated chemical oxygen demand (COD), biological oxygen demand (BOD), and a host of refractory compounds, dye-laden wastewater is challenging to manage, representing a formidable task. The infiltration of organic dyes into aquatic environments not only amplifies water coloration but also obstructs sunlight penetration, depletes dissolved oxygen reserves, and disrupts ecological balance.^156,157^ For example, a 2D zinc­(II)-based MOF was produced under solvothermal conditions and demonstrated notable photocatalytic activity for organic dye degradation without requiring any photosensitizer or cocatalyst.^158^ The catalyst’s stability and reusability were key parameters in evaluating its performance. Recyclability tests showed that, after five consecutive cycles, the MOF retained 91.7% degradation efficiency for RhB, with negligible loss in activity, reflecting strong long-term stability. In contrast, for MO dye, the efficiency decreased to 60.3% after five reuse cycles. UV–vis analysis revealed a primary absorption band near 280 nm, and the estimated band gap was 3.282 eV, suggesting that the relatively narrow band gap contributed to enhanced light absorption and improved photocatalytic performance.

In another work, a 2D MOF-5/ZnMgAl LTH hybrid nanocomposite was synthesized via a facile hydrothermal route, having unique features.^159^ The developed sample was recorded with a photocatalytic efficiency of 95.1%, twice that of the MOF-5 nanocomposite for MB when exposed to UV–visible light radiations within 125 min, as demonstrated (Figure 11a). The as-prepared photocatalyst showed superior reusability for up to 5 successful runs with minimum loss in the catalytic performance. The degradation efficiency achieved was reported to be twice pure MOF-5. The elimination of MB dye was found to follow a pseudo-first-order kinetic model, with rate constants close to 1, confirming the model’s applicability. Figure 11b,c depicts the proposed photocatalytic mechanism, where UV–visible light induced electron excitation by VB to CB, forming e–h^+^ pairs. The VB holes acted as strong oxidants, either directly degrading dye molecules or generating ^•^OH radicals, while conduction band electrons reduced oxygen species to form H_2_O_2_. Besides this, photocatalytic degradation of methyl orange was demonstrated with 2D Cd/Co-based MOFs fabricated using hydrothermal conditions, under the influence of UV radiation.^160^ The Cr­(VI) reduction performance of free H_2_L, P25, BUC-66, and BUC-67 within UV exposure was compared. BUC-66 and BUC-67 achieved 98% and 99% reduction within 30 min, respectively, while free H_2_L and P25 showed only 24% and 46% efficiency. Recyclability tests confirmed that BUC-66 and BUC-67 retained high activity for Cr­(VI) conversion and MO breakdown after five cycles. Both MOFs demonstrated significant photocatalytic performance within Cr­(VI)/organic dye systems. Under UV irradiation for 30 min, complete reduction of Cr­(VI) was achieved; however, deterioration efficiencies for MO reached 85% for BUC-66 and 100% for BUC-67.

**Figure 11:**
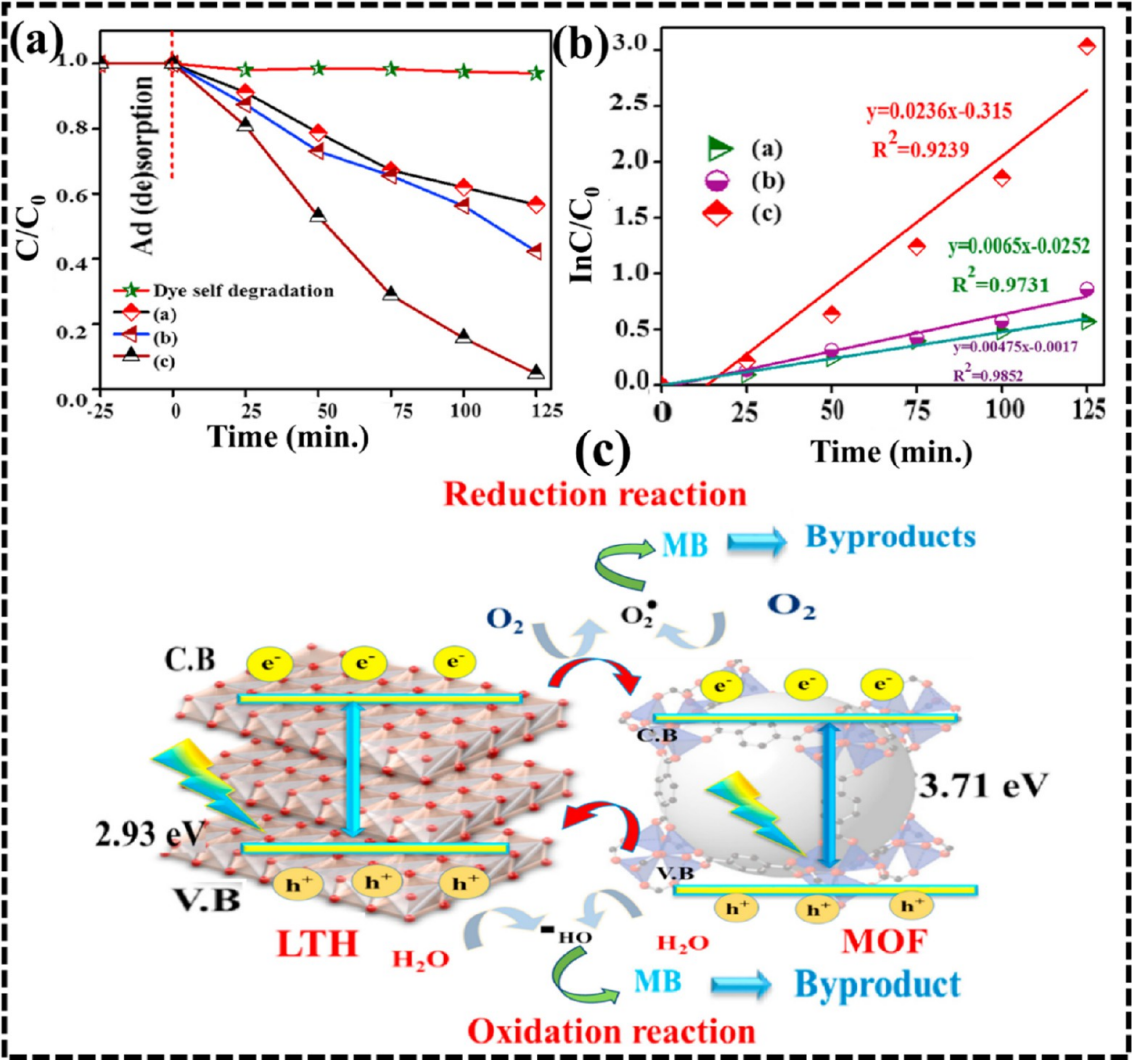
(a) Photocatalytic degradation of methylene blue (MB) dye (a) MOF-5, (b) LTH, and (c) the MOF-5/LTH hybrid. (b) Corresponding pseudo-first-order kinetic plots were reported for (a) MOF-5, (b) LTH, and (c) the MOF-5/LTH hybrid. (c) Schematic illustration of the proposed photocatalytic mechanism for the MOF/LTH hybrid. Reproduced with permission from Elsevier (license no. 6075300189666).^159^

### 4.3 Antibiotic Degradation

Rapid industrial development worldwide has led to a surge in contaminants being released into aquatic ecosystems, significantly affecting water quality despite water being vital for the survival of all living organisms.^136^ Antibiotics, important emerging organic pollutants, are extensively used for treating humans and animals. However, their widespread use has raised concerns about antibiotic resistance. Tetracycline (TC) and oxytetracycline hydrochloride (OTC) are prominent broad-spectrum antibiotics used to combat bacterial infections and as feed additives to promote growth in aquaculture.^161^ TC and OTC are both widely used in animal husbandry as veterinary drugs. They serve two primary purposes: preventing infections and promoting growth in livestock. However, less than 30% of the administered antibiotic dose is absorbed by the animals; the remaining portion is excreted as feces and released into the environment.^162,163^ Antibiotic pollution has emerged as a critical concern in highly populous nations where agricultural demands are intensified.^136^ Employing advanced drug elimination techniques presents a promising strategy for minimizing the duration of prolonged dialysis sessions.^164^ For instance, Wu and their colleagues prepared a 2D/2D FeNi-LDH/BMNSs nanomaterial using an in situ semisacrificial template approach.^165^ PL, TPC response, and EIS Nyquist plots were analyzed to evaluate the samples. The weakest PL intensity noted in FeNi-LDH/BMNSs-1 indicated the most efficient separation of photogenerated carriers. Without H_2_O_2_, e^–^–h^+^ reassembly was enhanced, suggesting that charge carriers participated in the photo-Fenton process, thereby improving their separation. FeNi-LDH/BMNSs-1 showed the elevated TPC density, reflecting the abundance of exposed active sites. The photo-Fenton catalytic capacity of all samples was evaluated via TC-HCl degradation under light exposure. Degradation efficiency followed the trend: FeNi-LDH NSs (4.20%) < FeNi-LDH/BMNSs-0.5 (63.45%) < FeNi-LDH/BMNSs-5 (85.51%) < FeNi-LDH/BMNSs-1 (95.76%), with FeNi-LDH/BMNSs-1 achieving the highest TOC removal rate (52.80%). The FeNi-LDH/BMNSs + H_2_O_2_ + Vis system demonstrated superior stability and recyclability, retaining distinct structural peaks after cycling, confirming structural integrity.

In another study, a novel 2D/2D S-scheme heterojunction Ni-MOF/BiOCl was developed using a self-assembly method for the effective removal of TC.^82^ The degradation efficacy was elevated from 50.82% to 95.78% in 180 min upon an increased concentration of 0.1Ni-MOF/BiOCl from 10 mg to 100 mg. For degradation of TC, two pathways were proposed based on LC–MS study as shown in Figure 12. As demonstrated in Figure 12, the complex toxic TC pollutant was photoconverted into simpler and innocuous products by processes such as dehydroxylation, demethylation, deamination, ring opening, and decarboxylation. Besides this, the ·OH and ·O_2_^®^ radicals played a major role in the degradation process proved via ESR study. Furthermore, the hydrothermal method was employed by Hu and team to fabricate defect-free 2D-MOF membranes via LDH and utilized for effective rejection of up to 98.5% of TC.^166^ The pristine PVDF support membrane showed low tetracycline (TC, 0.01 mM) removal efficiency (8.5%) despite its prominent H_2_O penetrability, attributed to its large pores indicated via a greater MWCO of 827 kDa. In contrast, surface modification with LDH and 2D-MOF layers significantly enhanced TC rejection. The 2D-MOF membrane obtained markedly higher TC removal (98.5%) than the LDH membrane (40.2%) at neutral pH, while maintaining a greater-water flux of 622.9 L m^–2^ h^–1^ bar^–1^. Furthermore, the 2D-MOF membrane maintained over 90% removal efficiency over a broad pH spectrum and TC concentrations, indicating strong removal capability. It also exhibited better antifouling properties and durability in prolonged filtration, offering potential for developing exceptional 2D membranes for purifying water and antibiotic-infected water treatment.

**Figure 12:**
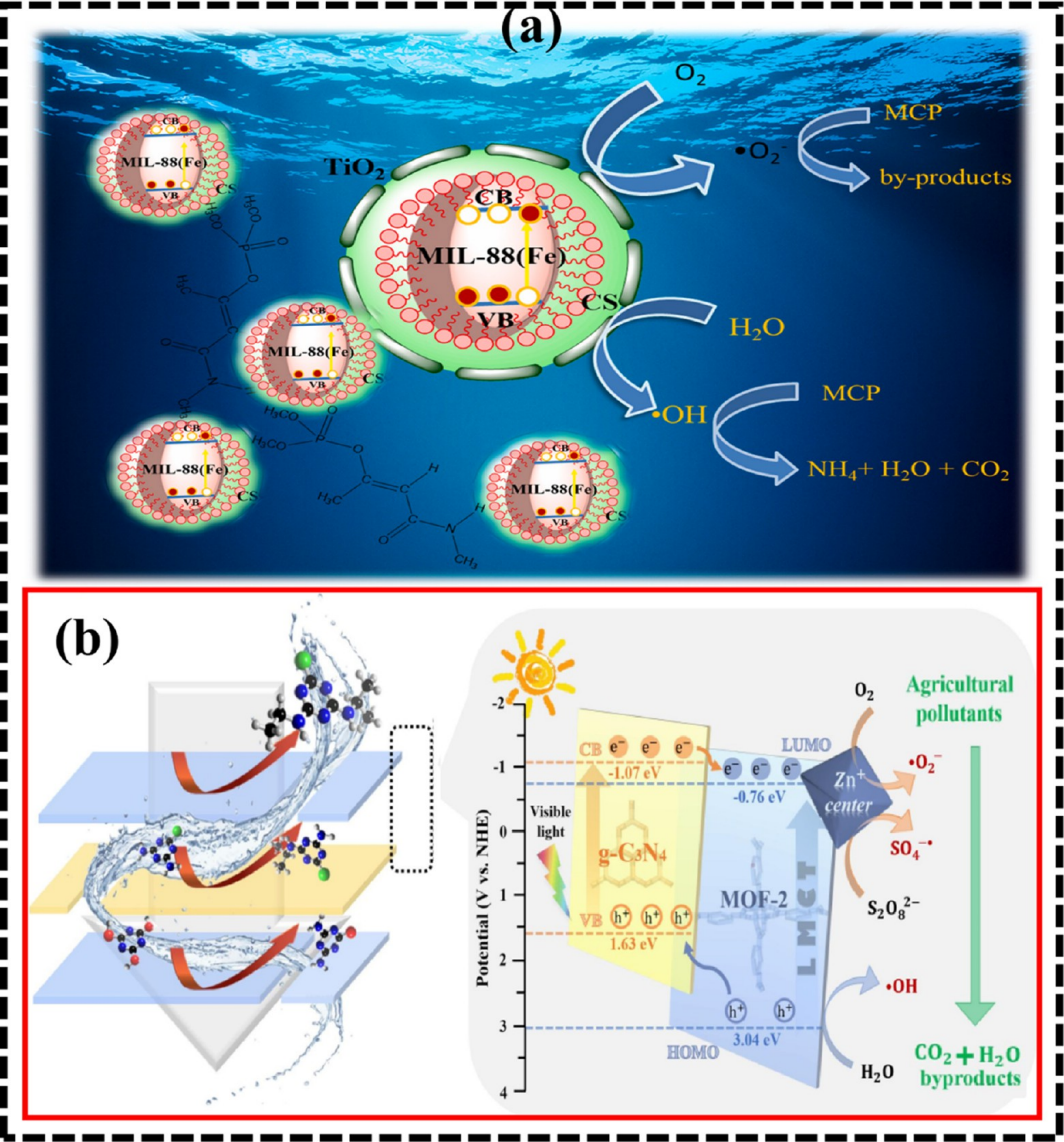
(a) Schematic illustration of the electron–hole transfer in TCS@MOF during MCP oxidation under visible light (30 min, 50 mg/L dosage, 20 mg/L MCP, pH 6 ± 0.2, 350 W, 0.15 mW/m^2^, 24.5 °C). Adapted with approval from Elsevier (license no. 6075761315150).^32^ (b) Proposed mechanism for agricultural pollutant removal by 2D heterostructure membranes via persulfate-mediated photocatalysis.. Reproduced with permission from Elsevier (license no. 6075770014011).^167^

### 4.4 Pesticide Degradation

Driven by the world’s growing community and the resulting food problem, the agriculture field is actively looking for ways to increase crop yields to satisfy the rising demand for grain.^167^ However, this requirement invariably calls for the regular use of agricultural pesticides. To shield fruits and vegetables from the threats posed by pests, insects, and diseases, pesticides are extensively utilized in modern agricultural practices.^168,169^ Consequently, pesticide residues are prevalent in most produce as these chemicals are used both during cultivation and after harvesting, raising significant health concerns.^170–172^ Thus, various techniques have been employed to effectively eliminate pesticides from edibles.^173,174^ Due to extensive surface area and exceptional porosity, MOFs are indispensable materials that are highly effective in pesticide removal. Vigneshwaran and team used the solvothermal technique of synthesis to generate a 2D/2D TiO2/MIL-88­(Fe) that showed improved photocatalytic efficiency in degrading organic pesticide monocrotophos (MCP).^32^ Under identical experimental conditions, MCP exhibited the highest degradation efficiency compared to other pollutants. The removal performance of MCP by TCS, MIL-88­(Fe), and TCS@MOF followed the chain: TCS < MIL-88­(Fe) < TCS@MOF, with increasing efficiency over irradiation time under visible light. The TCS@MOF system achieved a photocatalytic elimination efficacy of 97.79% within 30 min. Figure 12a illustrates the effective separation of electron–hole pairs and MCP mineralization via energetic radicals in the Vis/TCS@MOF system. A correlative analysis was conducted on MCP deterioration using different composites under various conditions.

In another work, there was a vacuum-assisted self-assembly process involving orderly interlayer nanochannels of MOF-2 and g-C_3_N_4_ nanosheets.^167^ The 2D heterostructure responsible for enhanced response range for light radiations along with reduced recombination, contributed to enhanced photocatalytic performance (Figure 12b). The 2D membranes were systematically evaluated for durability and recycling throughout the long run. The MF/CN-2 membrane maintained stable flux under varying pH conditions due to strong interlayer interactions. In pure water, ATZ removal reached approximately 98%, while elimination proficiency exceeded 93% in river water. After five reuse cycles, the membrane sustained high removal performance, with ATZ removal remaining above 80%. The preservation of membrane structure contributed to the consistent performance. Flux drop in the nanocomposite was mitigated by strong interlayer interactions, with MF/CN-2 maintaining a stable flux of 23.6 L m^–2^ h^–1^ bar^–1^ over 5 h. Ultimately, the 2D heterostructure membrane, having exceptional endurance across a broad spectrum of pH and pressure conditions, underscores its significant potential for sustained and effective cleanup of agricultural contaminants, contributing to enhanced integrity of water protection. To illustrate recent developments in the field, (Table 3) summarizes other representative studies involving 2D MOF-derived photocatalysts.

**Table 3:** Overview of Fabrication Methods and Photocatalytic Properties of 2D MOF-Derived Materials, Detailing Their Specific Surface Area, Light Response Range, and Effectiveness against Specific Pollutants along with Evidence of Durability

S. No.	2D MOF material	preparation method	surface area (m^2^ g^–1^)	light absorption range	targeted pollutant	activity	stability evidence	ref.
1	Ni-MOF/NiB-1	solvothermal method	171	visible region	Cr(VI)	96%	PXRD	^153^
2	BUC-66 and BUC-67	hydrothermal	-	390 and 490 nm, respectively	Cr(VI)	98% and 99%, respectively	PXRD, TGA	^160^
3	CuMOF-Ti-3	in situ solvothermal method	432.2	UV–vis region	Cr(VI) reduction into Cr(III)	99.9%	ICP-Mass	^175^
4	KLU-10 and KLU-11	hydrothermal method	1.77 and 26.87	visible region	MO	97.59% and 95.61%, respectively	TGA, PXRD	^176^
5	g-CN@Ni-bpy	one-pot solvothermal	-	visible light	MG	97.7%	-	^90^
6	Zn-MOF@ZnO and Cd-MOF@ZnO	ultrasonication-assisted hydrothermal method	36.458/39.54	visible region	methyl violet (MV)	90.1%, 92.5%, respectively	FESEM, PXRD	^177^
7	Fe_3_(HITP)_2_	co-precipitation method	260.2	UV–vis	TC, tinidazole and norfloxacin	96.7%, 90%, and 85%, respectively	XRD, XPS, TEM and ICP	^178^
8	MOF-BiOCl/MoS_2–3_	solvothermal method	-	visible spectrum	TC	90%	XRD	^131^
9	TCS@MOF	solvothermal method	935	visible spectrum	MCP	97.79%	XRD	^32^
10	MOF 1 and MOF 2	facile solvothermal approach	239.42 and 157.78	visible spectrum	CO_2_ reduction	365.59 and 276.89 μmol g^–1^	XRD, XPS	^53^
11	MOF-2/g-C_3_N_4_	vacuum-assisted self-assembly	-	visible region	ATZ	98%	SEM	^167^
12	Ce-TCPP and La-TCPP	solvothermal approach	462.8 and 434.6	visible light active	H_2_O_2_ production	21.02 and 79.75 μmol/g/h	FTIR, XRD	^91^
13	BMO/ZTP	in situ self-assembly	91	visible spectrum	H_2_ production	10900.94 μmol/g/h	SEM, XRD, FTIR	^85^
14	Co-MOF/p-g-C_3_N_4_	electrostatic self-assembly	21.8206	visible region	H_2_ evolution	73.42 μmol h^–1^	-	^94^
15	NC80	in situ growth	50	visible light active	H_2_ and N-BBA production	8.5 and 4.6 mmol g^–1^ h^–1^	XRD, FTIR	^130^

## 5 Conclusion and Future Perspective

2D MOFs have emerged as a significant subclass of MOF catalytic materials, providing various advantages in the field of environmental remediation, mainly for wastewater treatment. Unlike their 3D counterparts, 2D MOFs have ultrathin layered structures that endow them with a remarkably large surface-to-volume ratio, improved accessibility of catalytic sites, as well as reduced charge migration pathways. These characteristics are particularly favorable for different photocatalytic applications, as they allow improved absorption of light, effective charge isolation, and stronger interaction with contaminant molecules. As this review highlights, 2D MOF-based nanocomposites have displayed substantial efficacy in the photodegradation of a wide range of environmental pollutants, including toxic heavy metals, organic dyes, pharmaceutical remains like antibiotics, and persistent pesticides. These alterations in 2D MOFs allow for precise functionalization of both metal nodes as well as organic linkers. This structural flexibility enables the incorporation of different functionalities, heteroatoms, or cocatalysts into the structure, significantly boosting the selectivity and reactivity of these catalytic materials toward specific target water pollutants.

Moreover, designing 2D MOFs tailored to specific targets requires a rational understanding of how their structural features dictate photocatalytic behavior. The choice of metal nodes controls the redox potential and active-site distribution, while the functionalization of organic linkers can modulate band gap energy, charge transport pathways, and pollutant adsorption affinity. Layer thickness and interlayer spacing also play decisive roles in charge separation and mass transport efficiency. Therefore, optimizing parameters such as coordination environment, pore size, and linker conjugation offers strategic avenues to engineer 2D MOFs for selective and efficient photocatalysis.

Additionally, the modified pore size and directional channels in 2D MOF nanocomposites help to enhance the mass transfer and adsorption of pollutants, both of which are critical to catalytic efficiency. Various synthesis methods and modification strategies have been developed to enhance the photocatalytic performance and stability of 2D MOF materials. These include bottom-up fabrication approaches, liquid-phase exfoliation methods to obtain ultrathin nanosheets, and postsynthetic alterations such as doping with either metal or nonmetal species, hybridization with other photoactive semiconductors or carbon-based catalytic materials, and construction of different heterojunctions. Such alterations not only improve light-harvesting efficiencies or charge carrier mobility but also boost the structural robustness under photocatalytic operating environments. Despite their various advantages, several key challenges remain before 2D MOF-based photocatalytic materials can be extensively deployed in real-world environmental applications. Thus, the following topics should be the focus of future research.

- Various MOFs, mainly in aqueous or acidic/basic medium suffer from structure collapse or the leaching of ligands, which restricts their long-term efficiency. Future studies should focus on improving the photocatalytic performance of MOF-based catalysts through enhancing structural integrity and resistance to degradation by incorporating chemically stable blocks, rigid linkers, and stable supports.
- Designing MOF materials through various modification strategies, like the integration of particular functional groups in a way to increase their selectivity, being helpful in targeting desired pollutant molecules. Introducing electron-donating/withdrawing groups, acidic/basic catalytic sites, and redox-active moieties into the structure can enhance the isolation of charge carriers, improve the affinity of various pollutants, and molecular recognition, enabling more precise as well as effective catalytic systems.
- A major limitation lies in scaling up the fabrication of 2D MOFs without compromising their structural integrity as well as functionality. Advancing the development of low-cost, environmentally benign, scalable synthesis techniques to synthesize multifunctional 2D MOFs that can break down pollutants effectively via performing adsorption and catalysis at the same time, thus optimizing their performance in real-world applications.
- Ensuring the reusability of 2D MOF-based catalytic materials is vital for their practical deployment. Future studies should focus on understanding the photocatalytic degradation pathways and developing different strategies to prevent deactivation. Robust 2D MOFs that retain structural as well as catalytic efficacy after different catalytic cycles are vital to realizing sustainable remediation technologies.
- More precise evaluations of MOF-based catalyst efficiency, offering deeper insights into their practical applicability via modeling real-world conditions through systematic variations in reaction parameters, including pH, temperature, mass and the influence of supplement materials. Systematic findings under these conditions will give a deeper understanding of efficiency limitations and thus help to optimize the reaction environment for large-scale practical applications.
- Expanding the applicability of MOFs across various fields beyond water treatment, as not all MOFs are commercially accessible. Future research should emphasize cost-effective fabrication, resource-efficient metal selection, and scalable synthesis methods to evaluate their cost and market availability along with cost checks on integrating metal into MOFs to enhance production.
